# The Oldest Highlands of Mars May Be Massive Dust Fallout Deposits

**DOI:** 10.1038/s41598-020-64676-z

**Published:** 2020-06-25

**Authors:** J. Alexis P. Rodriguez, Eldar Noe Dobrea, Jeffrey S. Kargel, V. R. Baker, David A. Crown, Kevin D. Webster, Daniel C. Berman, Mary Beth Wilhelm, Denise Buckner

**Affiliations:** 1 Planetary Science Institute, 1700 East Fort Lowell Road, Suite 106, Tucson, AZ 85719-2395 USA; 2 Department of Hydrology & Atmospheric Sciences, University of Arizona, Tucson, AZ 85721 USA; 3 NASA Ames Research Center, Moffett Field, CA 94035 USA; 4 University of North Dakota, Department of Space Studies, Grand Forks, ND 58202 USA; 5 Blue Marble Space Institute of Science, 1001 4th Ave, Suite 3201, Seattle, WA 98154 USA

**Keywords:** Astronomy and planetary science, Geomorphology

## Abstract

The oldest terrains of Mars are cratered landscapes, in which extensive valleys and basins are covered by ubiquitous fluvial plains. One current paradigm maintains that an impact-generated megaregolith underlies these sediments. This megaregolith was likely largely generated during the Early Noachian (~4.1 to ~3.94 Ga) when most Martian impact basins formed. We examined the geologic records of NW Hellas and NW Isidis, which include this epoch’s most extensive circum-basin outcrops. Here, we show that these regions include widespread, wind-eroded landscapes, crater rims eroded down by several hundred meters, pitted plains, and inverted fluvial and crater landforms. These surfaces exhibit few fresh craters, indicating geologically recent wind erosion. The deep erosion, topographic inversions, and an absence of dunes on or near talus across these regions suggest that sediments finer than sand compose most of these highland materials. We propose that basin-impact-generated hurricane-force winds created sediment-laden atmospheric conditions, and that muddy rains rapidly settled suspended sediments to construct extensive Early Noachian highlands. The implied high abundance of fine-grained sediments before these impacts suggests large-scale glacial silt production and supports the previously proposed Noachian “icy highlands” hypothesis. We suggest that subglacial meltwater interactions with the sedimentary highlands could have promoted habitability, particularly in clay strata.

## Introduction

The cratered highlands of Mars include extensive ancient terrains that formed during the Noachian Period (~4.1 to ~3.7 Ga; ages herein based on Neukum chronology^1^). Some of the planet’s earliest geologic investigations, primarily based on Viking Orbiter images obtained during the 1970s, suggested that these mountainous areas consist of impact-generated megaregolith^2,3^. Higher resolution surface image and topographic data acquired during the last two decades revealed that these terrains also include extensive sedimentary deposits^4–7^.

The best documented of these sedimentary deposits are those that form many of the planet's intercrater plains. While some of these terrains might be lava fields, the recent finding that many consist of friable, wind-erodable materials indicates that they include vast areas of sedimentary origin^8^. Some investigations suggest that episodic floods during the Middle and Late Noachian (~3.94 to ~3.71 Ga)^9–11^ emplaced some of these plains materials under paleoclimatic conditions of long-term aridity^12,13^.

However, fluvial sedimentation during a “wet” anomaly is also thought to have contributed to their accumulation. The fluvial discharges during this period^14–21^ were short-lived (potentially <1 Ma)^22,23^ and imprinted on the cratered highlands’ vast systems of valley networks during the Late Noachian/Hesperian boundary (~3.71 Ga^19,20^).

**Figure 1:**
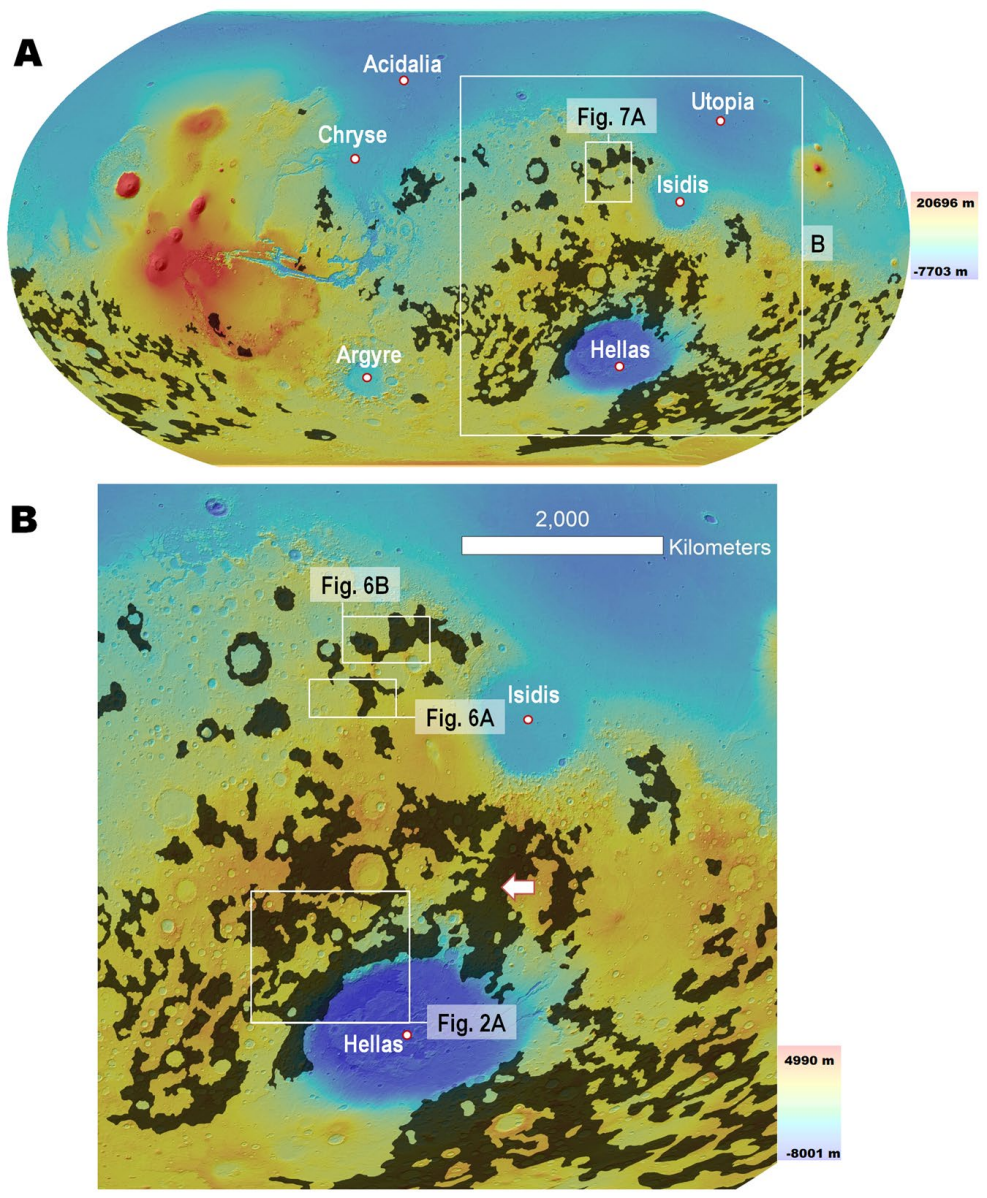
**(A)** Map showing the global distribution of Early Noachian terrains on Mars (dark areas). This map is a modification of the global geologic map by Tanaka *et al*.^7^, in which we merged the Early Noachian highland massif unit (eNhm) and the Early Noachian highland unit (eNh). We annotated the planet’s largest impact basins which, according to the geologic history reconstructed from the map^7^, formed during this epoch. Note that of these, only Hellas and Isidis exhibit extensive Early Noachian terrains along their margins. The locations and context for panel (B) and Fig. 7A are shown. **(B)** Zoom in on panel (A) showing the locations and context for Figs. 2A, 6A, B. The white arrow identifies Early Noachian highlands that are proximal to both Isidis and Hellas, and hence most likely contain ejecta materials from both impacts. **Panels (A)** and **(B)** are color-coded shaded-relief MOLA digital elevation models (460 m/pixel, credit: MOLA Science Team, MSS, JPL, NASA). We produced this figure using Esri’s ArcGIS^®^ 10.3 (http://www.esri.com/software/arcgis).

The stratigraphic record beneath these fluvial plains is thought to mostly consist of massive volumes of Early Noachian impact-generated megaregolith (~4.1 to ~3.94 Ga)^6^. This geologic superposition relationship is consistent with the finding that most of Mars’ impact basins >150 km in diameter formed^6,7^ during this epoch (i.e., >65 for Early Noachian, >15 for Middle Noachian, 3 for Late Noachian, 4 for Hesperian, and 2 for Early Amazonian^7^). These Early Noachian basins include the planet’s largest (i.e., Chryse, Utopia, Acidalia, Hellas, Argyre, and Isidis, Fig. 1). Hence, the fallout of clastic materials ejected by these impacts must have resulted in both the emplacement of enormous ejecta blankets around the impact basins and significantly contributed to the formation of a global megaregolith.

These impacts, however, could have triggered paleoclimates conducive to rainfall^24–26^, perhaps followed by episodic glaciation. The longevity of these periods of rainfall remains uncertain. Segura *et al*.^25^ considered possible transient wet periods lasting several centuries. However, other researchers hypothesize that the rainfall lasted several months to a few years^27^–^28^. Thus, it is conceivable that extensive Early Noachian glacio-fluvial and aeolian deposits covered large parts of the Martian megaregolith.

While the Middle and Late Noachian history of fluvial degradation led to the significant destruction of crater rims^9^, it did not planate these ancient cratered landscapes^29,30^. Thus, Mars retains extensive surface exposures of the materials that make up these extremely old highlands. The goal of this article is to determine whether the highland stratigraphy that lies buried beneath the Noachian intercrater plains contains a geologic record consistent with widespread sedimentation that happened as a consequence of the previously proposed impact-modified atmospheric and paleoclimatic conditions^24–28^. We find strong similarities between the sedimentary record preserved in NW Hellas and NW Isidis, suggesting a link between basin-forming impacts, consequential paleoclimate changes, and catastrophic sedimentary fallout and deposition.

## Evidence of an Early Noachian Depositional Upper Crust on Mars

In this section, we present the results of a geologic investigation (see Supplementary Materials for Methods and Datasets) of multiple study areas in NW Hellas and NW Isidis (Fig. 1B). These regions include the planet’s most extensive Early Noachian exposures as mapped by Tanaka *et al*.^7^. While other smaller exposures occur in SW circum-Chryse^7^ (Fig. 1A,B),  equivalent exposed stratigraphy appears to be absent around the Acidalia, Utopia, and Argyre basins, probably because it was largely stripped off and buried^7^ (Fig. 1A).

Because the study regions are proximal to these basins’ margins, they comprise zones of expected voluminous impact ejecta blanket deposition. Hence, they are ideal for assessing the nature of potential sedimentation under impact-modified atmospheric conditions and paleoclimates. Furthermore, because the areas include terrains as far apart as ~4,000 km, their investigation provides the opportunity to examine the geology of similar, yet geographically independent, impact basin margins.

### The Northwestern Hellas Region of Mars

The NW Hellas region includes the planet’s most extensive circum-basin Early Noachian terrains (Fig. 1). Extensive fluvial activity appears to have dominated erosion of these ancient highlands, producing vast depositional plains that mantle the interiors of numerous impact craters and most intercrater regions^7,31–35^. In this section, we present observations that are relevant to the sedimentology and origins of these deposits and the geologic materials comprising the highlands that the plains embay.

#### The Intercrater Plains: Wind Erodable, Fine-Grained Deposits

**Figure 2:**
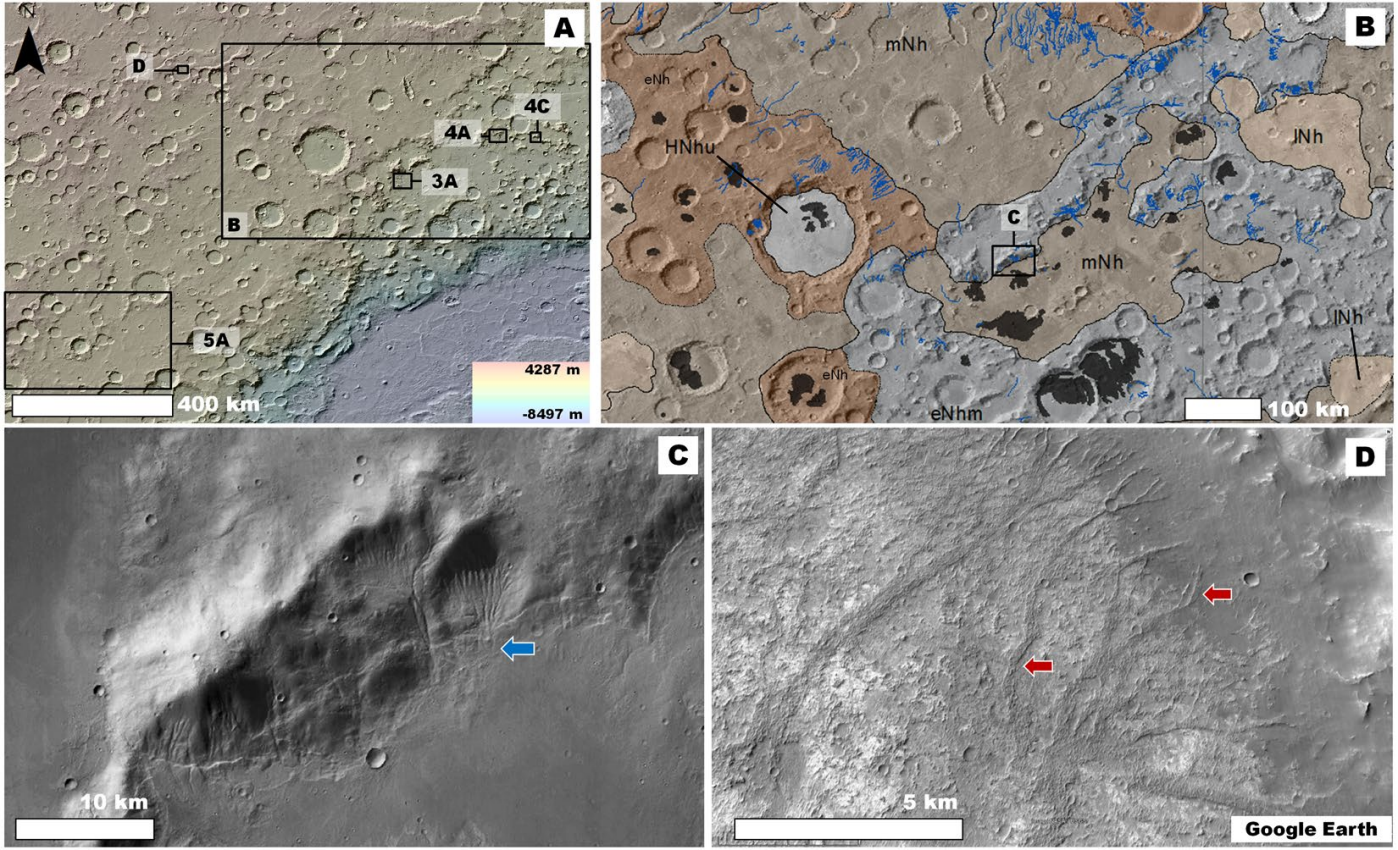
**(A)** View of northwestern and western Hellas centered at 27°28′S, 48°31′E. The image includes the context and locations of panel (B) and Figs. 3A, 4A,C, and 5A. The panel’s base is a color-coded shaded-relief MOLA digital elevation model (460 m/pixel, credit: MOLA Science Team, MSS, JPL, NASA). **(B)** View of an area of northwestern Hellas centered at 25°0′S, 55°27′E, which includes widespread pits (areas shaded dark) that occur within an Early and Middle Noachian regions. The geologic units were extracted from the global geologic map of Mars^7^, and include Hesperian and Noachian highland undivided unit (HNhu), the Late Noachian highland unit (lNh), the Middle Noachian highland unit (mNh), the Early Noachian highland massif unit (eNhm), and the Early Noachian highland unit (eNh). The blue lines trace the valley network channels, which we mapped in the region. The base image is part of a THEMIS Day IR (infrared) Global Mosaic (http://www.mars.asu.edu/data/, 100 m/pixel, credit: Christensen *et al*.^71^). **(C)** View centered at 25°14′31 S, 55°20′41″E showing some channels that dissect a promontory in an Early Noachian area (blue arrow). Note how the terminal regions of the channels abruptly end at their contact with the plains. **(D)** View centered at 20°6′S, 42°49′E showing part of an inverted tributary channel system exhumed from within crater interior plains (red arrows). The downslope direction is towards the bottom left. **Panels** **(C)** and **(D)** are parts of a CTX mosaic (6 m/pixel, credit: NASA/JPL. The license terms can be found at pds-imaging.jpl.nasa.gov/portal/mro_mission.html). We produced this figure using Esri’s ArcGIS^®^ 10.3 (http://www.esri.com/software/arcgis).

**Figure 3:**
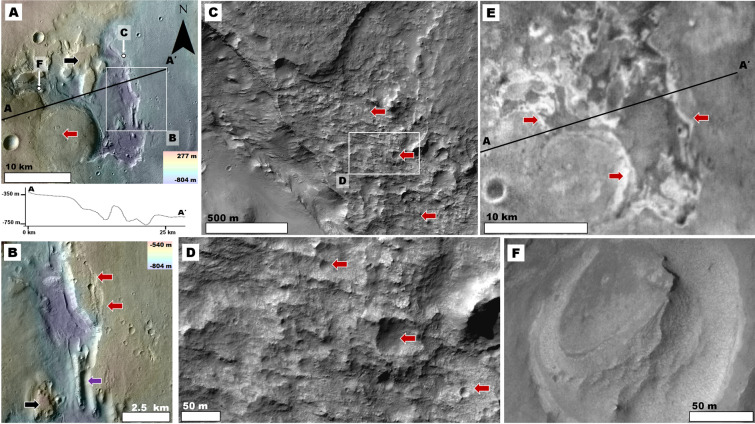
**(A)** View of pit centered at 25°35′S, 55°28′E. See Fig. 2A for context and location. The red arrow identifies a partly exhumed circular mesa, which we interpret as an inverted crater fill, excavated because it contained materials that are more resistant to erosion than its surrounding plains. The elevation profile shows rim-to-floor depths of ~200 m within the pit. The white arrows identify the center locations of panels (C) and (F). **(B)** Close-up view of panel (A) showing the pit’s eastern margin. The red arrows and the purple arrow, respectively, identify areas of shallow and deep dissection. The black arrow identifies a mesa with a surface that has a similar elevation to the pit’s adjoining plains. **Panels (A)** and **(B)** are parts of a color-coded shaded-relief MOLA digital elevation model (460 m/pixel, credit: MOLA Science Team, MSS, JPL, NASA) over parts of a CTX mosaic (6 m/pixel, credit: NASA/JPL. The license terms can be found at pds-imaging.jpl.nasa.gov/portal/mro_mission.html). **(C)** Close-up of part of the pit’s eastern margin showing grooves that dissect almost the entire relief of the scarp (red arrows). **(D)** Close-up view of the panel (C) showing an absence of craters with pristine rims and preserved ejecta blankets at multimeter and decameter scales on the scarp. The red arrows identify numerous small craters that have dissected margins. **Panels (C)** and **(D)** are part of HiRISE image ESP_026492_1540 (50 cm/pixel, credit: NASA/JPL/University of Arizona (https://www.uahirise.org/media/usage.php)). **(E)** View of the pit showing that its interior scarps exhibit relatively high thermal inertia (red arrows). This panel is part of a THEMIS nighttime IR (infrared) global mosaic (http://www.mars.asu.edu/data/, 100 m/pixel, credit: Christensen *et al*.^71^). **(F)** Close-up of part of the pit floor showing characteristic smooth surfaces. In general, pit floors show no residual collapse, mass wasted materials, or boulder-rich deposits. HiRISE image ESP_038373_1540 (50 cm/pixel, credit: NASA/JPL/University of Arizona (https://www.uahirise.org/media/usage.php)). We produced this figure using Esri’s ArcGIS^®^ 10.3 (http://www.esri.com/software/arcgis).

Our observations show that the NW Hellas plains include numerous pits (Fig. 2A,B), which are generally several tens of kilometers across and a few hundred meters deep (e.g., Fig. 3A). These surface depressions lack evidence of a collapse origin such as knobby floors and extensionally faulted margins (e.g., Fig. S1). Instead, their peripheries include  contacts along which the transitions from the plains are characterized by grooves incised to different depths (e.g., Fig. 3B). These observations point to progressive surface dissection playing a key role in the formation of the pits. The presence of interior mesas with surface elevations matching those of the surrounding plains (black arrows in Fig. 3A,B) further supports an origin due to differential erosion. The materials along the pits’ flanks generally lack boulders (e.g., Fig. 3C,D) and are bright in THEMIS nighttime IR images (e.g., Fig. 3E), indicating that fine-grained, weakly cemented sediments make up the bulk of the materials exposed along their scarps^36^. The pits’ sparse distribution reflects an origin due to erosional disturbances localized to some areas of the plains. The erosion resulted in the significant removal and distant transport of the sediments, as indicated by a lack of nearby overland surface deposition. Based on these observations, we interpret that wind erosion was the primary landscape modifying mechanism leading to pit formation. On Earth, similar wind-sculpted landscapes exist (e.g., Fig. S2).

When observed at decameter scales, the pits’ flanks exhibit grooves that breach the margins of most of their superposed craters, including those smaller than ~100 m across (e.g., Fig. 3D). This observation suggests that some of the erosion was geologically recent, extending support towards the proposed prominent role of wind in pit excavation. Since these dissected craters lack rocky ejecta blankets, the ejecta was likely composed of fines that were eroded away during surface dissection. Hence, the proposed young aeolian erosional phase removed both surface and impact excavated materials. The hypothesized aeolian origin of the pits implies that — at least to their equivalent depths — a fine-grained sedimentary composition dominates the plains stratigraphy. The inferred sedimentary makeup of these plains is consistent with our observation that the pit floors appear smooth and lack appreciable residual boulders (Fig. 3F).

#### Fluvial Slurry Flows as a Source of the Intercrater Plains: The Break of a Paradox?

Numerous investigators argue that Late Noachian fluvial sedimentation produced the intercrater plains of NW Hellas^37–39^. However, because the lowermost reaches of this region’s valley networks abruptly terminate at their contact with intercrater plains, the geomorphic evidence of fluvial sedimentation as their emplacement mechanism remains ambiguous (Fig. 2C).

**Figure 4:**
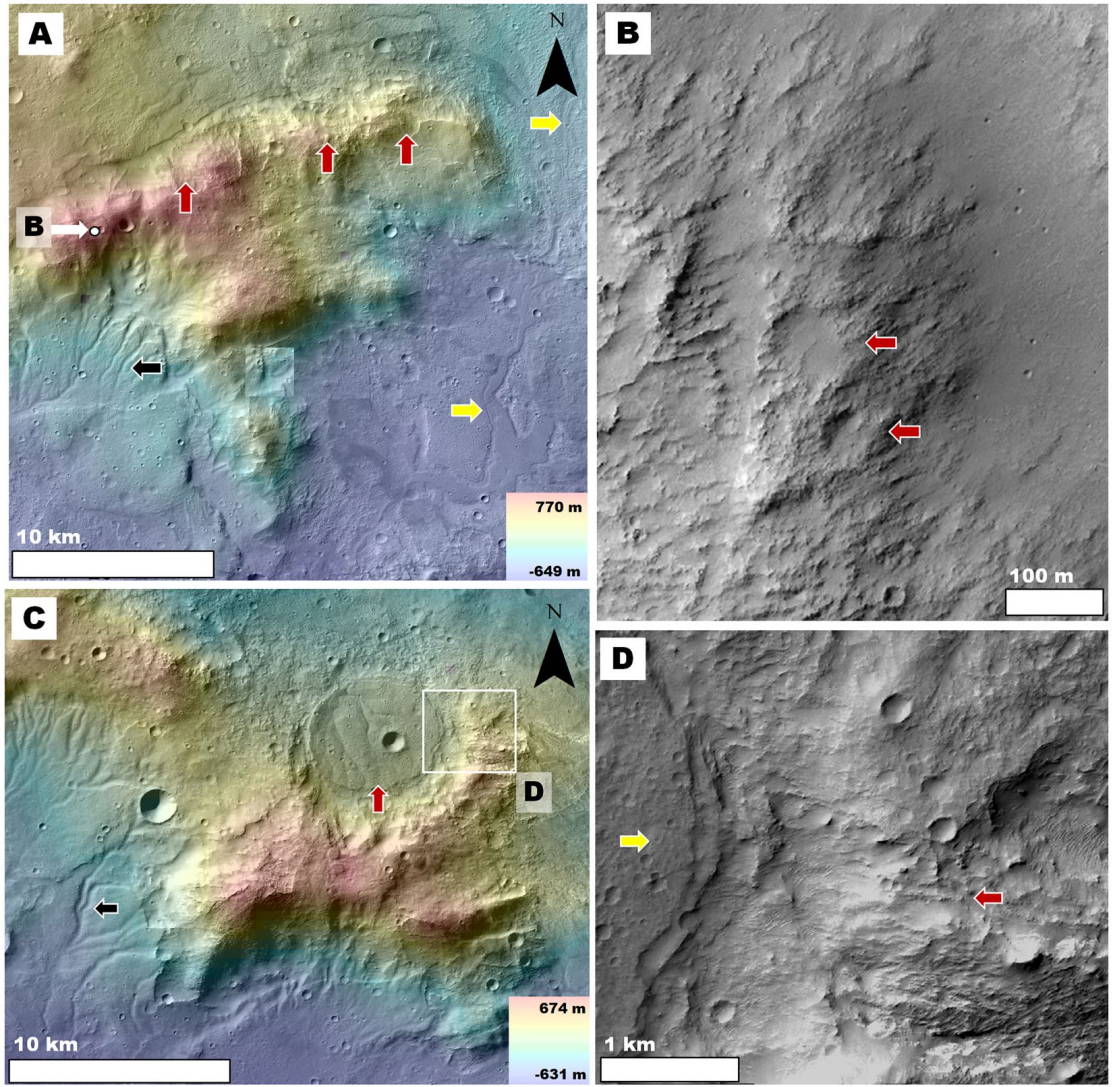
(**A**) View of an Early Noachian promontory centered at 23°30′S, 59°37′E. See Fig. 2A for context and location. The promontory exhibits a deeply eroded northern flank (red arrows) and some of its talus shows channels (black arrow). The surrounding plains include inverted fluvial topography (yellow arrows). The white arrow’s head identifies the center of panel (B). The image is a composite of a color MOLA digital elevation model (460 m/pixel, credit: MOLA Science Team, MSS, JPL, NASA) draped over a CTX mosaic (6 m/pixel, credit: NASA/JPL. The license terms can be found at pds-imaging.jpl.nasa.gov/portal/mro_mission.html). **(B)** Close-up view of part of one of the erosional surfaces shown in panel (A). The view shows widespread grooves, some of which modify the margins of relatively small craters (red arrows). HiRISE image ESP_036487_1560 (25 cm/pixel, credit: NASA/JPL/University of Arizona (https://www.uahirise.org/media/usage.php)). **(C)** Early Noachian highland exposure centered at 23°31′S, 61°33′E in which differential erosion led to the excavation of an impact crater fill, which has a surface marked by channels (red arrow). See Fig. 2A for context and location. Some of its talus materials also exhibit channels (black arrow). The image is a composite of a color MOLA digital elevation model (460 m/pixel, credit: MOLA Science Team, MSS, JPL, NASA) draped over a CTX mosaic (6 m/pixel, credit: NASA/JPL. The license terms can be found at pds-imaging.jpl.nasa.gov/portal/mro_mission.html). **(D)** Close-up view showing a margin of the inverted crater (yellow arrow) and its adjoining highlands. Note that, in contrast with the crater fill materials, the surrounding highlands are significantly grooved (red arrows). The image is a CTX mosaic (6 m/pixel, credit: NASA/JPL. The license terms can be found at pds-imaging.jpl.nasa.gov/portal/mro_mission.html). We produced this figure using Esri’s ArcGIS^®^ 10.3 (http://www.esri.com/software/arcgis).

We have identified some locations where valleys transition into topographically inverted channel deposits (e.g., Figs. 2D, 4A, S3). Topographic inversion of channels on Earth typically occurs because fluvial sorting concentrates relatively coarse sediments within their floors and depositional fans. These materials become preserved as remnant ridges  when their adjacent fine-grained overbank floodplain deposits (clay and silts) are removed through aeolian erosion^40^. Similar processes likely formed the channel inversion on Mars^40–42^.

We propose that the burial of significant parts of former fluvial systems’ distal reaches beneath overbank floodplain materials can explain the general absence of depositional features at the valley networks terminal areas, thereby reinforcing the previously postulated fluvial origin of the plains^37–39^. We observe that the valley networks of NW Hellas have regional geographical extents (Fig. 2B). These valleys are locally eroded and discontinuous. However, their upper and lower reaches, typically situated in mountains (mostly crater rims) (e.g., Fig. 2B) and their nearby plains (e.g., Fig. 2C), respectively, are generally well preserved. The retention of these channel segments permits accurate determinations of the fluvial networks’ extents. We found that the relatively short valley lengths (tens of kilometers) constrain the origin of their sediments to local mountainous sources (e.g., Figs. 2C,4A,B). These observations imply that a transition from erosional to depositional fluvial sedimentary environments occurred in short distances. Similar fluvial settings on Earth occur where floods erode and ingest underlying, unconsolidated or weakly cemented fine-grained substrates, thereby generating slurry flows that can form broad plains^42^.

The short-range transition of fluvial run-off morphologies into depositional plains, along with the identification of integrated tributaries exposed by erosion, together explain the poor valley network integration in NW Hellas and strengthen the case for the previously hypothesized prominent role of regional fluvial deposition. More importantly and in the context of the investigation of the regional Early Noachian stratigraphy, it also suggests that this fluvially dissected highland stratigraphy, just like it is apparent in the region’s intercrater plains, also consists of fine-grained sediments.

#### The Early Noachian Highlands: Vast, Cratered, Sedimentary Units

We have identified surfaces in highland promontories that exhibit tens of meters of lost relief due to localized erosion (Fig. 4A,B). These surfaces lack extensive boulder beds. Instead, they are marked by grooves a few tens of meters wide and broader, along with similarly oriented excavated ridges (Fig. 4B), suggesting an origin by wind erosion. These surfaces are bright in THEMIS nighttime IR images (Fig. S4), indicating that the wind-erodable materials likely consist of weakly cemented, fine-grained sediments^36^.

A similarity between these highland erosional surfaces and those observed in the pit interiors points to youthful wind erosion – most impact craters several tens of meters across have dissected rims and lack ejecta blankets (Fig. 4B). We counted the number of relatively fresh craters (i.e., those retaining some ejecta blankets) in the area shown in Fig. S5A. We found that there are very few of these (Fig. S5A), which is consistent with a recent history of wind erosion. The modeled surface age using these counts is ~28 Ma (Fig. S5B). This age has large error bars and was performed over an area of just 156 km^2^. Hence, we interpret its significance as indicating that erosion is young, even ongoing. An *in-situ* investigation using images from the Opportunity Rover supports our interpretation^43^. The investigation shows that small craters that formed over sedimentary rocks in Meridiani Planum appear to have been degraded by geologically recent wind activity.

Furthermore, as observed in the plains, these proposed wind-eroded areas also exhibit exhumed inverted relief, including circular mesas connected to nearby channel systems (e.g., Fig. 4C). By analogy to the sedimentology of inverted channels^40^, this association indicates that relatively coarse fluvial deposits might be abundant within these circular mesas.

**Figure 5:**
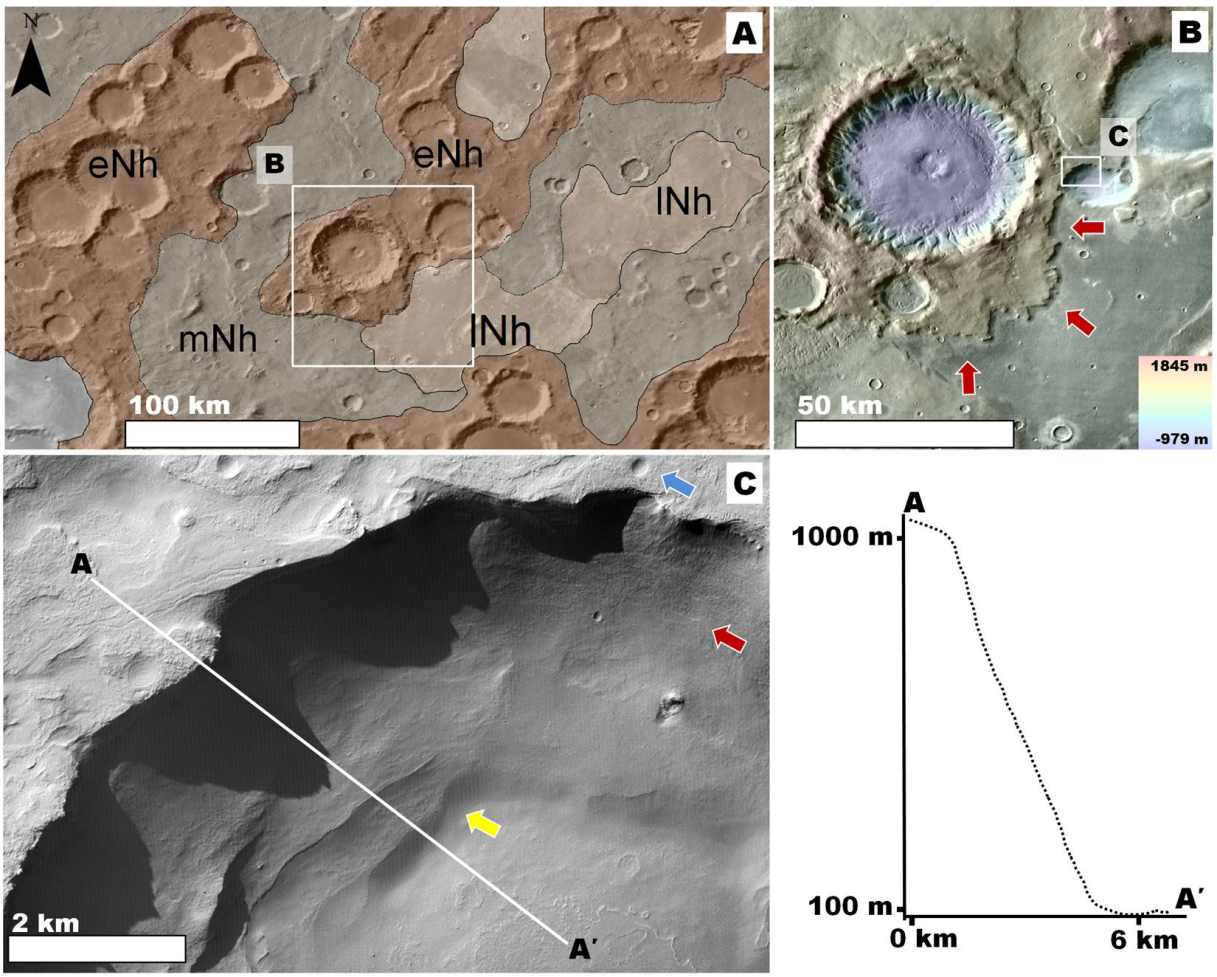
(**A**) View of western Hellas, which includes extensive Noachian cratered highlands centered at 34°43′S, 38°46′E. The geologic units were extracted from the global geologic map of Mars^7^, and include the Late Noachian highland unit (lNh), the Middle Noachian highland unit (mNh), and the Early Noachian highland unit (eNh). See Fig. 2A for context and location. The base image is part of a gray shaded-relief MOLA digital elevation model (460 m/pixel, credit: MOLA Science Team, MSS, JPL, NASA). **(B)** Close-up view of panel (A) showing part of an Early Noachian terrain, which is scarp-bounded to the south and east (red arrows). The image is a composite of a color MOLA digital elevation model (460 m/pixel, credit: MOLA Science Team, MSS, JPL, NASA) draped over a part of a THEMIS Day IR (infrared) Global Mosaic (http://www.mars.asu.edu/data/, 100 m/pixel, credit: Christensen *et al*.^71^). **(C)** Close-up of the scarp in a zone where it bounds the rim of an ancient crater. A relatively high albedo layer (blue arrow) overlies a darker and slightly stratified deposit (red arrow). Enhanced retreat in these darker materials relative to the overlying bright rocks undercut the scarp, pointing to voluminous debris fallouts. However, the scarp front (yellow arrow) lacks significant talus deposits. The image is part of a CTX mosaic (6 m/pixel, credit: NASA/JPL. The license terms can be found at pds-imaging.jpl.nasa.gov/portal/mro_mission.html). We produced this figure using Esri’s ArcGIS^®^ 10.3 (http://www.esri.com/software/arcgis).

Evidence of deep wind erosion on highland promontories indicates that fine-grained sediments probably make up most of the region’s upper crust. However, a difficulty in furthering the investigation of the stratigraphy of the NW Hellas Noachian highlands stems from the rarity of plateau strata exposed throughout their surfaces. We found an exceptional Early Noachian landscape where a prominent scarp reveals hundreds of meters of the terrain’s underlying geologic materials (Fig. 5A,B). This region occurs within the highlands that flank Hellas, positioned where substantial thicknesses of boulder-rich ejecta materials were emplaced during the basin’s formation. However, instead of a bouldery substrate, which would be consistent with ejecta fallout, the scarp exposes ~700 m of slightly stratified materials that lack bouldery outcrops (Fig. 5C).

At some locations, the scarp exhibits cantilever cliffs, indicating that there has been retreat. However, the absence of talus suggests that large quantities of the shed debris have been removed. The removal of these materials was likely due to wind erosion. If interstitial ice was abundant, then talus volume losses could have also been driven by sublimation. The role of wind erosion in the removal of the lithic component of the talus indicates that it is made up of sand or finer sediments, because coarser materials cannot be transported by wind. This proposition is consistent with the fact that the talus surfaces are dark (cold) in THEMIS nighttime IR images (Fig. S6), indicating that they likely consist of unconsolidated fine-grained sediments^40^.

Collectively, these observations suggest that a fine-grained highland-forming layer extends down to at least half a kilometer below the surface. This inference is consistent with the general absence of bouldery strata in the stratigraphy exposed within the plains’ pits, which suggests that the region’s overall impact history did not commonly result in the excavation of the underlying  boulder bearing ejecta substrates.

### The Northwestern Isidis Region

Might the fine-grained upper crustal deposits of NW Hellas and their environments of deposition have been unique in the Early Noachian? To address this question, we analyzed other Martian regions in similar geologic settings to those of NW Hellas. The highlands peripheral to the Isidis basin comprise the planet’s only other significant occurrences of circum-basin terrains of Early Noachian age (Fig. 1). Some of these terrains are north of Hellas and south of Isidis and are within a few hundred kilometers of the margins of both basins. Hence, their stratigraphy likely combines ejecta from both impacts and other deposits associated with these impacts.

**Figure 6:**
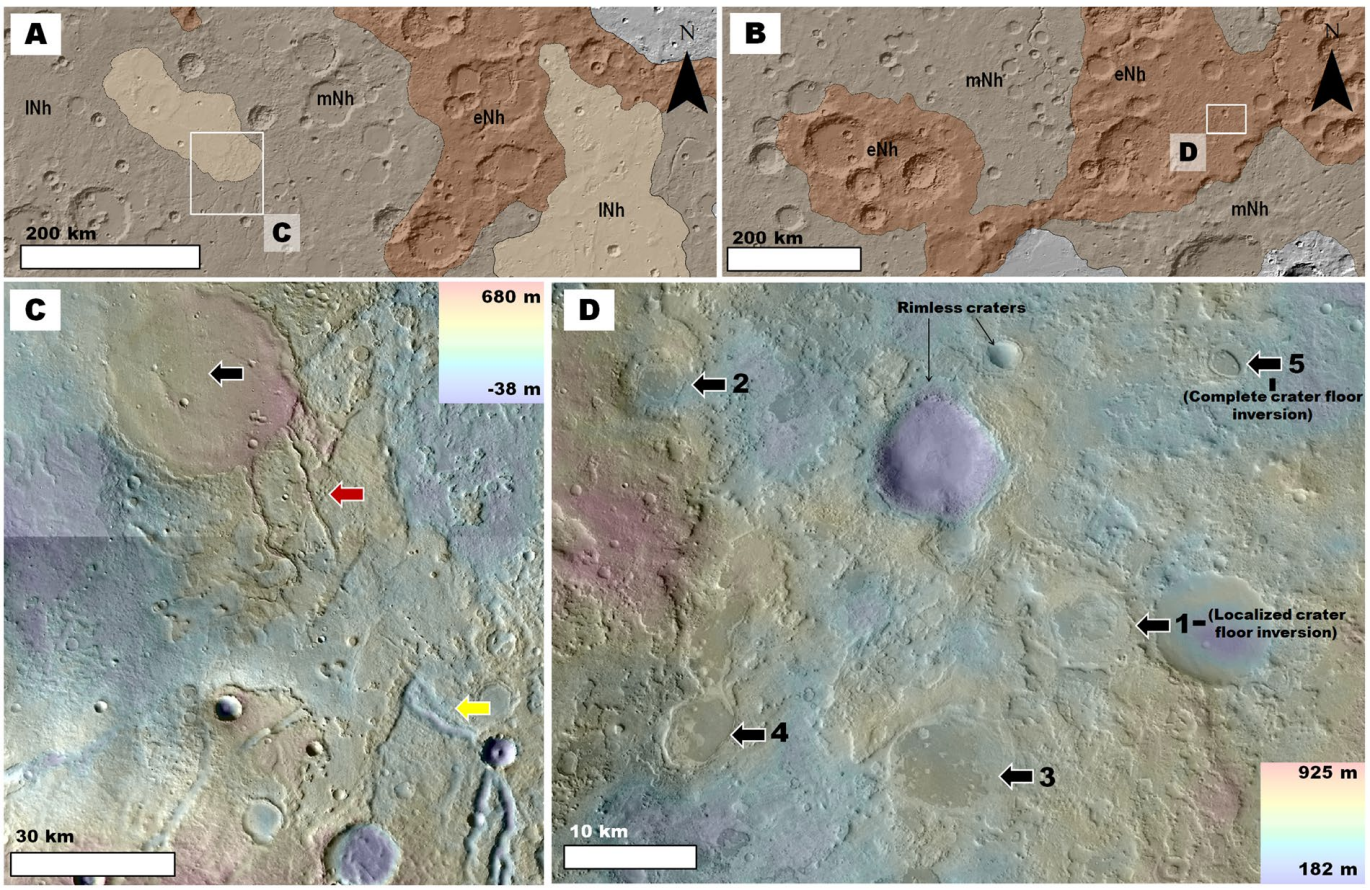
Views of circum-Isidis Noachian highlands located in eastern Terra Sabaea (**A**) and northeastern Arabia Terra (**B**). The geologic units were extracted from the global geologic map of Mars^7^, and include the Late Noachian highland unit (lNh), the Middle Noachian highland unit (mNh), and the Early Noachian highland unit (eNh). See Fig. 1B for the location and context of these panels. The base image is part of a gray shaded-relief MOLA digital elevation model (460 m/pixel, credit: MOLA Science Team, MSS, JPL, NASA). **(C)** Zoom in on panel (A) showing plains areas affected by extensive surface erosion in eastern Terra Sabaea. The view includes inverted crater fill (black arrow) connected to inverted channels (red arrow), which are located ~20 km downstream from the lower ends of regional valley networks (yellow arrow). The image is a combination of a color MOLA digital elevation model (460 m/pixel, credit: MOLA Science Team, MSS, JPL, NASA) draped over a CTX mosaic (6 m/pixel, credit: NASA/JPL. The license terms can be found at pds-imaging.jpl.nasa.gov/portal/mro_mission.html). **(D)** Zoom in on panel (B) showing plains areas affected by extensive surface erosion in NE Arabia Terra. The black arrows labeled 1–5 identify examples of impact crater topographic inversion due to the removal of the region’s plains.

Consequently, we highlight and discuss the circum-Isidis highland areas that are located in eastern Terra Sabaea and NE Arabia Terra (Figs. 1B, 6A,C), both of which are further than a thousand kilometers away from Hellas and likely offer independent stratigraphic records (Fig. 1). Like the Hellas highlands^32^, the circum-Isidis regions have long been considered parts of a major area of dust and sand activity^44,45^. An early remote-sensing investigation in these regions identified the presence of a several-hundred-meter thick mantle over the circum-Isidis cratered highlands of NE Arabia Terra and interpreted it to be composed of mostly dust^46^.

We find that the geologic characteristics indicative of the presence of thick, fine-grained, upper crustal deposits in NW Hellas are accentuated in the investigated circum-Isidis terrains. For example, both regions of the planet exhibit intercrater plains that include exhumed, topographically inverted craters, and channels. However, in the circum-Isidis terrains, the intercrater plains’ removal is widespread and not confined to the interior of pits (Fig. 6), as is the case in Hellas (Figs. 2B,3A). These observations are consistent with the presence of fine-grained, plains-forming Noachian materials.

**Figure 7:**
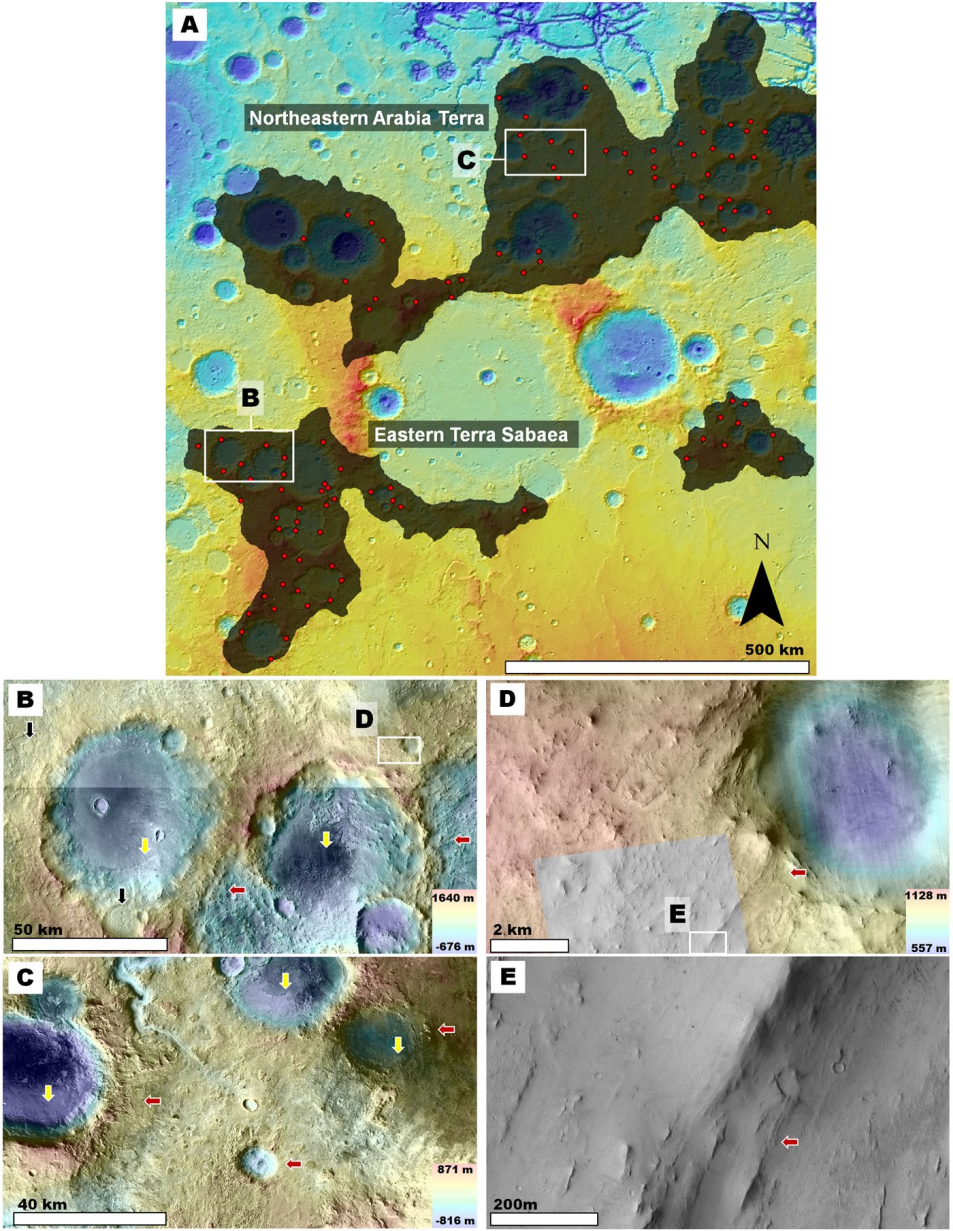
(**A**) View of the Early Noachian highlands (shaded areas) of eastern Terra Sabaea and NE Arabia Terra as extracted from the global geologic map of Mars^7^. The red dots identify numerous (but not all) crater rim areas that exhibit deep breaches with grooved floors. Note that many of these breaches incise craters’ entire rim reliefs. The base image is part of a gray shaded-relief MOLA digital elevation model (460 m/pixel, credit: MOLA Science Team, MSS, JPL, NASA). **(B** **and C)** Zoom in views on panel (A). Impact craters within Early Noachian terrains that exhibit significantly eroded rims. The black arrows identify inverted craters exhumed from within the rim-forming materials of a crater in panel (B). The red arrows point to grooved surfaces forming the floors of crater breached rim areas. Most of these craters have floors, which are not significantly eroded and locally show inverted topographic margins (yellow arrows). **Panels** **(B)** and (**C**) are composites of a color MOLA digital elevation model (460 m/pixel, credit: MOLA Science Team, MSS, JPL, NASA) draped over a CTX mosaic (~6 m/pixel, credit: NASA/JPL. License terms can be found at pds-imaging.jpl.nasa.gov/portal/mro_mission.html). **(D and E)** Zoom in views on panel (B). These views show the retention of grooved surfaces at hectometer and decameter scales (red arrows). Note that these surfaces generally lack craters with pristine rims and ejecta blankets. **Panel** **(D)** is a composite of a color MOLA digital elevation model (460 m/pixel, credit: MOLA Science Team, MSS, JPL, NASA) draped over a CTX mosaic (~6 m/pixel, credit: NASA/JPL. License terms can be found at pds-imaging.jpl.nasa.gov/portal/mro_mission.html). The grayscale image section on the forefront is part of HiRISE image PSP_007438_2000 (25 cm/pixel, credit: NASA/JPL/University of Arizona (https://www.uahirise.org/media/usage.php)). **Panel** (**E**) is a close-up view of HiRISE image PSP_007438_2000 (25 cm/pixel, credit: NASA/JPL/University of Arizona (https://www.uahirise.org/media/usage.php)).

**Figure 8:**
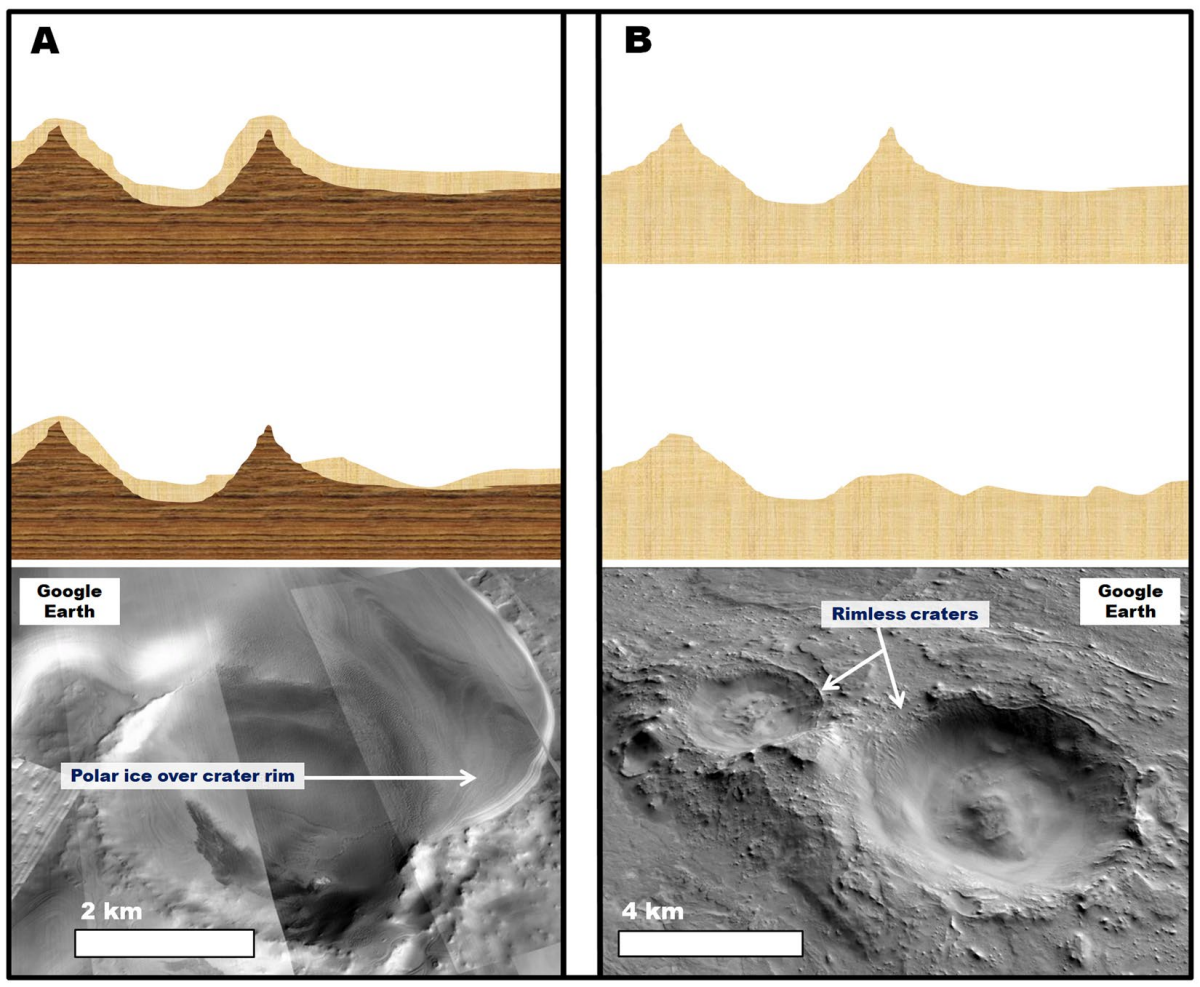
(**A**) **Top and middle panels:** Sketches showing the landscape resulting from the partial removal of a lithologically weak mantle unit (light orange area) from over an impact crater excavated into a competent basement (brow area). In this case, the mantle's erosion exhumes part of the crater rim to form a ridge that preserves the crater's topography. **Bottom panel:** View of a crater rim that has been partly exhumed from beneath an icy mantle in Planum Australe (80° 8′10.91″S, 96°39′40.41″E). The image is part of a CTX mosaic (~6 m/pixel, credit: NASA/JPL). **(B) Top and middle panels:** Sketches showing the landscape evolution resulting when erosion affects an impact crater excavated into a deep, lithologically weak, sedimentary unit. In this case, the entire crater's relief might be eliminated. **Bottom panel:** View of two craters NE Arabia Terra, which we interpret to superpose a fine-grained upper crustal deposit. The craters have largely removed rims (25°43′55.88″N, 60°16′53.40″E). The image is part of a CTX mosaic (~6 m/pixel, credit: NASA/JPL).

A key observation is the presence of craters, which regardless of diameter, have rims that exhibit substantial to complete relief losses in breached zones (Fig. 7). The rim breaches are extensively grooved and some reach or exceed the crater floor depths (red arrows in Fig. 7B,C). These craters regionally co-exist with numerous inverted crater fills (black arrows in Fig. 6B,C; black and yellow arrows in Fig. 7B,C). These observations suggest the wind removal of substantial volumes of surface and subsurface materials, including a significant fraction that was impact uplifted and exposed from within the highlands. These materials, we propose, do not form mantles over the cratered landscape^46^ (Fig. 8A) but instead, comprise the plateau-forming stratigraphy over which these craters occur (Fig. 8B).

We performed counts of fresh craters (i.e., those retaining some ejecta blankets) on the rims of the cluster of large craters shown in Fig. 7B (see Fig. S7 for the boundaries of the counts area). These craters exhibit inverted crater fills exhumed from within their rims (black arrows in Fig. 7B) and from within parts of their crater floors (yellow arrows in Fig. 7B). These observations suggest that large amounts of weakly cemented or loosely aggregated sediments were removed by wind. We obtained a modeled age of ~45 Ma (i.e., Late Amazonian (Fig. S7)). We chose to perform the counts on the rims because, when compared to the adjoining plains, they must have been relatively unaffected by deposition and are hence more representative of the timing of erosion. This young age is consistent with the proposed aeolian erosion extending into the Late Amazonian; however, it does not rule out more lengthy erosion. Upon examination at hectometer/decameter scales, the breached surfaces show grooves lacking pristine superposed craters (Fig. 7C), pointing to a wind erosional history that locally occurred to recent times.

## Discussion

### Early Noachian Sedimentation Peripheral to Impact Basins

Our results indicate that the materials characterizing the highlands that flank the NW areas of Hellas and Isidis are fine-grained sediments. In this section, we discuss the sedimentology of these materials, the geologic mechanisms that could have produced them, and how their accretion history during the Early Noachian led to the formation of the highlands.

#### The Deposits’ Grain Size and Sorting

The emplacement of the fine-grained Early Noachian upper crustal highland deposits, described in both circum-Hellas and circum-Isidis, buried and postdated the basins’ ejecta blankets and melt that were produced during the impact-forming events (see Supplementary Materials on The Production of Impact Melt Associated with the Formation of Hellas). Our observations suggest that the fine-grained materials are wind erodable, which constrains their dominant sizes to sand or finer grains. The specific grain size of these materials is difficult to further narrow down using remote-sensing image data, but some geomorphic observations, which we present in this section, are relevant and provide critical constraints.

Our examination of talus, deposited from localized scarp degradation within the Early Noachian highlands study areas, reveals a general absence of bedforms (Figs. 5C, S8), suggesting that their dominant grain size is that of finer-than-sand sediments. Dunes in these areas form scattered deposits and some exhibit fractures consistent with their materials being lithified paleo-dunes (e.g., Fig. S8B). These geologic characteristics suggest significant starvation in the regional sand supply, despite the presence of talus. Furthermore, we have not observed dune fields or wind streaks that source at the plains’ pits or the highland erosional surfaces. This observation indicates that wind transported, and might be transporting, the removed sediments by suspension and that, therefore, fine grains (finer than sand: clay and silt sized dust) likely characterize their bulk sedimentary make-up. Some of the lithified fine-grained materials could break down into mudstone and siltstone boulders, pebbles, and sand; however, these coarse materials would undergo similar wind degradation over time.

The magnitude of the crater rim breaches and removal points to erosional depths that reached several hundred meters to a few kilometers. When considered in the regional extents of these materials, this observation implies the general absence of sediments coarser than sand (ranging from pebbles to boulders) intermixed with the fines. If coarse-grained materials had been present in significant volumes, wind would have produced widespread deflation surfaces with coarse-grained lags capping the fines, thereby hindering their long-term removal from the highlands’ fine-grained stratigraphy. Deflation lags of just a few pebbles thick — somewhat thicker in rougher relief — is sufficient to shield underlying fines^47^.

#### Possible Geologic Sources of the Fine-grained Sediments in Early Mars

Our observations suggest that vast volumes of sedimentary rocks were present within the Martian upper crust during the Early Noachian. These materials could have had diverse geologic origins during both the Early Noachian and the pre-Noachian (a time period lasting several hundred million years^6,7^).

Little is known about the pre-Noachian geologic record; however, it has been observed that it includes large numbers of buried impact craters^6,7^. These units’ deposits and  interbedded impact craters might overlie a clay layer as much as ~10 km thick, which could have formed as a primordial basaltic crust that was substantially altered beneath a short-lived steam or supercritical atmosphere^48^. Hydrothermalism due to volcanism and/or impact heating could have also contributed to the alteration of these, or younger, basaltic rocks into clays^49^.

If such a primordial atmosphere developed on Mars, its progressive condensation could have generated a planet-wide hydrosphere. A combination of  the resulting hydrologic activity with impact (see Supplementary Materials on Impact Formation of Fines), volcanism, and wind processes most likely resulted in prolonged erosional/depositional cycles. These cycles would have produced voluminous sedimentary layers, which would have formed the pre-Noachian stratigraphy above the potential primordial clay-rich zone.

In the presence of a faint, young sun^50^, the planet’s hydrosphere could have generated widespread glaciers. On Earth, glaciation may be the most prodigious process responsible for the generation of silt^51^ (see Supplementary Materials on Glacial Formation of Silt). As Mars probably lacked the tectonic renovation of Earth, the materials could have been retained and accumulated to form significant parts of the planet’s early stratigraphy.

Furthermore, if the planet’s broad physiography persisted relatively unchanged during immense timescales, then the perseverance of global wind patterns could have resulted in long-term depositional sinks. Loess, volcanic ash, and fine-grained airborne ejecta would have accumulated into thick sequences under conditions of rapidly varying climate.

The global geologic map of Mars^7^ concludes that both volcanic activity and asteroidal impacts were prominent geologic processes during the Early Noachian. The potential of Early Mars’ glaciation (AKA the “icy highlands hypothesis") has been previously explored^52–54^; however, there are no documented glacier valleys dating back to the Early Noachian and older times. This apparent absence might be the consequence of extensive burial and impact-induced changes in topography, which would have obliterated most of the glacier evidence that could be recognizable from orbit.

#### Early Noachian Impacts and Episodic Catastrophic Fallout Sedimentation

Our results indicate that during the Early Noachian, thick layers— up to several hundred meters— of silt and dust were emplaced to form widespread upper crustal deposits. The apparent absence of significant coarse sediments within these materials suggests a history of size-sorting and deposition through atmospheric processes.

While the specific nature of fallout sedimentation remains uncertain, we can provide constraints and use these to develop a working hypothesis. An important constraint concerns the age of the fine-grained materials. To determine the Early Noachian age of the study regions, Tanaka *et al*.^7^ used the cumulative crater densities for all unit occurrences through a global crater database^55^, which consists of all craters with diameters >1 km.

We surveyed the circum-Isidis study areas using high-resolution image datasets and found that most of the craters with diameters >1 km in these regions exhibit evidence of wind-breached rims (Fig. 7A shows a significant sample of breached rim zones). Hence, nearly the entire population of large craters must have formed on the fine-grained deposits, which in turn indicates that these materials were emplaced during the Early Noachian.

Furthermore, our survey reveals a lack of crater rims made of Isidis bouldery ejecta blanket materials, which presumably underlie the fine-grained sedimentary deposits. Thus, the deposition of these materials must have occurred before the regional crater population formed, bringing the conditions that led to its deposition to just after, or temporally close to, the Isidis impact.

The massive character of deposition is consistent with an origin due to catastrophic atmospheric fallout, implying the presence of enormous volumes of fines in the atmosphere. We attribute the development of the sediment-laden atmosphere to extremely powerful winds created by basin-forming impacts, which captured fines that existed at the Martian surface and near-surface at the time. Pre-existing fines likely existed within both sorted and unsorted substrates (e.g., potentially debris flow deposits, glacial loess and moraines, marine and lacustrine sediments, volcanic ash, and impact regolith and ejecta blankets).

The formation of the major basins on Mars must have triggered massive, regional and global winds. Geologic and modeling observations indicate that tornado force winds probably formed streaks around various craters, each ~20 km across^56^. The effectiveness of these winds to capture and transport sediments might be gauged by the 2015 M7.8 earthquake in Langtang, Nepal, and the ensuing consequential ice and rock avalanche^57^. This event generated winds exceeding 300 km/hour (equivalent to an EF5 tornado) that scoured away an entire village of stone slab buildings (slab size commonly 15 ×15 ×30 cm) probably in less than one minute— leaving barely a trace of foundations— and knocked down a forest. The winds were triggered by gravitational settling of the avalanche-entrained rock and ice; similar and greater surface scouring by winds likely occurred on Mars in our hypothesized scenario.

Subsequent to initial impacts, atmospheric heat disturbances would have been caused by widely distributed impact melt and hot rocks (see Supplementary Materials on The Production of Impact Melt Associated with the Formation of Hellas). We have not assessed the wind speeds and longevity that would result during the cooling of these materials, but violent, sustained winds plausibly could have lasted several months to a year or more (especially over the basin and proximal ejecta), similar to the cooling times of thick lava flows.

The capacity of winds to scour sediments from the surface and hold them in suspension will increase with atmospheric density, which the basin-forming impacts would have likely boosted. Koeberl and Ivanov^58^ investigated impacts into Earth’s oceans during the Cryogenian, a geologic period when earth was covered by a global ice sheet. Their results confirmed that a huge amount of water vapor and radiatively active contaminants would have been ejected into the stratosphere by Chicxulub-size impacts. The likely presence of a northern ocean^59^ and extensive ice sheets^52–54,59^ during Early Mars make these investigations especially relevant. A Hellas basin-forming event may have been three orders of magnitude larger than the terrestrial Chicxulub event, which would have an even more amplified effect on Mars (a planet of 1/4 the area of Earth), causing transient atmospheres nominally many bars or tens of bars. Further, we note that the Noachian stratigraphy forming the Mars’ targets for basin-forming impacts may have contained abundant crustal volatiles^48,60,61^ (e.g., ice as well as salt hydrates, phyllosilicates, and carbonates). Impact-induced devolatilization of these materials could have also increased the atmospheric density.

The atmospheric fallout of the vast volumes of suspended sediments would have occurred as the winds lost speed and as the atmosphere cooled. A likely consequence of the subsequent cooling of a water vapor-rich atmosphere is the development of transient rainy paleoclimatic conditions^24–28^. We hypothesize that the rainfall could have effectively accelerated the removal rates of suspended sediments, resulting in catastrophic sedimentary fallouts in the form of muddy rains and the formation of voluminous muddy substrates over the Martian highlands. Hence, aeolian fallout — first of dry dust, and then of muddy rains — could have generated the Early Noachian-forming fine-grained sedimentary materials.

### Astrobiological Significance

While further work is required to establish the compositional diversity of the fine-grained upper crustal formations we document in this article, previous work shows that they contain numerous clay outcrops^61^. During the Noachian, when a globally integrated hydrosphere likely existed on Mars^7^, these formations could have provided widespread, thick, clay-rich substrates. Astrobiologically, these clay and water-rich environments could have potentially facilitated the emergence of life through the concentration and spreading of complex organics delivered by meteorites or produced *in-situ*, and then preserved any remaining putative molecular biosignatures.

Life on Earth emerged prior to ~3.7 Ga^62^, which falls within the Early Noachian Epoch of Mars, the age of the deposits we investigate here. At this time, the inner Solar System experienced a lengthy blitz of asteroidal impacts known as the Late Heavy Bombardment (LHB, ~4.1 to ~3.8 Ga)^63^. Influx peaks of carbonaceous material during this period could have facilitated the concentration and spreading of meteorite-delivered organics within the clay formations. Petrological studies of meteorites show that these organic materials include amino acids^64^, as well as various sugars^65^ and lipids^66^ required for life’s emergence. During the LHB, carbonaceous meteorites would have delivered organics to the surfaces of all the inner Solar System’s planets.

Yang *et al*.^67^ suggest that clay-rich geologic environments could have played a role in the origin of life. To demonstrate this potential, they produced simulated clay hydrogels in the laboratory by mixing clays and ancient ocean water simulants. The simulated environment provides confinement for biomolecules and biochemical reactions that are important for the emergence of complex life-forming biochemical processes and the evolution of life before the development of a cell membrane. In early chemical evolution, separation from the external environment is essential for the development and enhancement of the molecules and reactions^67^ required for cellular life.

Furthermore, terrestrial clay-rich deposits are known to host some of Earth’s oldest fossils^68^, highlighting the potential of these formations for *in-situ* investigations. Additionally, organic preservation can be enhanced by sorption to clay mineral surface, aggregation with minerals, and/or occlusion^69^.

An intriguing consequence of the Noachian icy highlands hypothesis^52–54^ is that extensive subglacial rivers and lakes could have existed on Mars. Relative to those that were present at the surface, these bodies of water could have exhibited long-term stability towards climate change, as well as day-night and seasonal temperature fluctuations. Hence, where they capped clay-rich outcrops, sustained habitability might have resulted.

The astrobiological importance of the Early Noachian fine-grained upper crustal materials is not limited to the possibility that these materials might have reproduced some of the conditions that could have led to the emergence of life on Earth. There is also the possibility that at some locations where these Early Noachian clays were buried and heated geothermally, habitability has been sustained even to present times.

Some of the Early Noachian cratered terrains appear to be buried beneath the northern plains^6^. The surface of the northern plains contains widespread mud volcanoes, pointing to the presence of a large subsurface, unfrozen, mud-rich stratigraphy^70^. We propose that zones of these northern plains buried highlands that were located beneath the cryosphere and situated within the hydrosphere could have comprised the mud volcanoes’ source regions. Hence, Early Noachian impact-fallout related mudstones could have been extruded to form mud volcanoes, thus making accessible a potential record of long-lived and, perhaps, continuing habitability. The mud volcanoes, therefore, are potential astrobiological targets of signicant importance (see Supplementary Materials on Case for Astrobiological Exploration).

## Conclusions

Most of the Martian impact basins formed during the Early Noachian, but only the peripheries of Hellas and Isidis retain highland terrains that date back to this geologic epoch. Our investigation into selected zones of these highland regions reveals evidence of deep wind erosion on extensive intercrater plain areas and numerous crater rims. The erosional depths point to the presence of a fine-grained sedimentary stratigraphy, reaching several hundred meters to over a kilometer in thickness. The general absence of bouldery outcrops suggests that the emplacement of these materials buried most of the basins’ ejecta blankets and they form the substrate on which these highlands’ cratered landscapes developed. The Early Noachian stratigraphy position of these materials between the basin formational events and the development of the cratered highlands suggests that deposition occurred during “brief” geologic time-scales.

We hypothesize that extremely powerful impact-triggered winds produced transiently densified, dust-laden, atmospheric conditions, and that the subsequent sedimentary fallout led to the formation of highly sorted, fine-grained, upper crustal sedimentary units. We propose that a newly considered geologic process for Mars could have played an important role in the proposed phases of upper crustal aggregation — muddy rains. Rainfall associated with impact-modified paleoclimatic conditions likely happened within dust-laden atmospheric conditions. The rain could have effectively removed large volumes of suspended dust, resulting in phases of mud precipitation and large-scale surface deposition.

A key implication of this hypothesis is that, prior to the impacts, the Early Noachian Martian surface and near-surface materials must have contained vast volumes of sedimentary fines. Numerous impact basins formed during the Early Noachian; hence, we predict the presence of depositional cycles within the highland stratigraphy, which might include layers deposited by the fallout of suspended sediments capped by the deposition of later fluvial sediments and glacial loess produced during sequences of impact-generated paleoclimatic “warmer and wetter” conditions. We highlight the importance of potential episodes of large-scale glaciation as a process to generate massive amounts of silt on early Mars. In this geologic scenario, the abundant fines supports the previously proposed Noachian “icy highlands” hypothesis. However, these materials could have had multiple other sources, including a pre-Noachian clay layer derived from an altered basaltic crust, lacustrine and marine silt, fluvial and debris flow deposits, volcanic ash, and impact ejecta. These deposits could have formed over the course of hundreds of millions of years during the pre-Noachian and Early Noachian. Consequently, they could have been exposed to a long history of hydrothermal alteration and other types of compositional modifications. Further mapping, spectral analysis, radar investigations, and future landers are required to establish the global extent and composition of these Martian units.

Our findings carry compelling implications towards understanding the histories of the Middle and Late Noachian. One is the possibility that fluvial activity occurring over these fine-grained materials resulted in short distance transitions to the areas of deposition, thereby providing a geologic scenario in which regionally poorly integrated valley networks formed in the presence of a very active hydrologic cycle on Mars. The other is that the impact events leading to the Noachian crater populations on the fine-grained crust could have potentially generated ice ages due to the atmospheric opacity by the loading of high dust contents.

The investigated Early Noachian landscapes, some ~4 Gyrs old, are so ancient that they allow us to examine the oldest water-related resurfacing processes in the Solar System. Earth retains minerals and metamorphosed rocks that date back to this time, but not landscapes. In contrast, our planet's oldest landscapes date back to just several tens of millions years ago. The deposition of the fine-grained crust happened possibly around the time when life existed on Earth and during which the inner Solar System was experiencing the Late Heavy Bombardment. Hence, it is probable that meteorites containing organic materials required for life could have been delivered to early Earth and Mars and exchanged between planets. The possible role of clays in both the emergence of life on Earth and the subsequent long-term preservation of biomolecules suggest that the investigated Early Noachian formations are important astrobiological targets.

## Supplementary information

Supplementary Materials.
