# Supplementary material for: The Oldest Highlands of Mars May Be Massive Dust Fallout Deposits
Source: Sci Rep. 2020 Jun 25;10:10347. doi: 10.1038/s41598-020-64676-z (PMC7316829; doi:10.1038/s41598-020-64676-z)
Supplement: Supplementary file 1 — Supplementary Materials. [file 41598_2020_64676_MOESM1_ESM.docx]

**Supplement to:**

**The Oldest Highlands of Mars May Be Massive Dust Fallout Deposits**

J. Alexis P. Rodriguez^1*^, Eldar Noe Dobrea^1^, Jeffrey S. Kargel^1^, V. R. Baker^2^, David A. Crown^1^, Kevin D. Webster^1^, Daniel C. Berman^1^, Mary Beth Wilhelm^3^, Denise Buckner^4,5^ (see author contribution statement for details).

*Correspondence to alexis@psi.edu

*^1^Planetary Science Institute, 1700 East Fort Lowell Road, Suite 106, Tucson, AZ 85719-2395, USA.*

*^2^Department of Hydrology & Atmospheric Sciences, University of Arizona, Tucson, AZ 85721, USA.*

*^3^NASA Ames Research Center, Moffett Field, CA 94035, USA.*

*^4^University of North Dakota, Department of Space Studies, Grand Forks, ND 58202, USA.*

*^5^Blue Marble Space Institute of Science, 1001 4th Ave, Suite 3201, Seattle, WA 98154, USA.*

**Table of Contents**

1. Methods and Datasets 2

2. Supplementary Figures 5

Figure S1 5

Figure S2 7

Figure S3 9

Figure S4 10

Figure S5 12

Figure S6 14

Figure S7 15

Figure S8 17

3. Impact Formation of Fines 19

4. Glacial Formation of Silt 23

5. The Production of Impact Melt Associated with the Formation of Hellas 26

6. A Case for the Future in-situ Astrobiological Exploration: Was the Early Noachian Fine-grained Crust an Important Source of Mud to the Northern plains Mud Volcanoes? 28

**1. Methods and Datasets**

**1.1. Morphologic and Analysis Mapping Approach**

To perform this investigation, we selected as study areas Early Noachian highlands situated along the margins of impact basins that also formed during this geologic epoch. To carry out our selection, we used the latest geologic map of Mars^1^ (Fig. 2A). The map shows that, while most of these basins appear to have formed during this geologic epoch, the highlands flanking Isidis and Hellas are unique in that they include the planet’s most extensive occurrences of Early Noachian, circum-basin highlands. Hence, we focused our geologic analysis on these regions of the planet (Figs. 1 to 7, S1 and S3), which are ideal for investigating the nature circum-basin sedimentation that followed major impacts during the Early Noachian. The images in the figures document key geologic features that are relevant to the formation and modifications of these ancient highlands. We performed the mapping of channels and pits, as displayed in Fig. 2B, at a scale of 1:500,000. Our primary analytical tool was the Environmental Systems Research Institute’s (ESRI) ArcGIS Desktop software (<http://www.esri.com/software/arcgis>).

**1.2. Datasets**

We utilized Mars Reconnaissance Orbiter (MRO) Context Camera (CTX, ~6 m/pixel) and High-Resolution Imaging Science Experiment (HiRISE; 25 cm/pixel*)* images and Mars Orbiter Laser Altimeter (MOLA) global Digital Elevation Model (DEM, ~460 m/pixel horizontal; ~30 cm vertical precision, <https://astrogeology.usgs.gov/search/map/Mars/GlobalSurveyor/MOLA/Mars_MGS_MOLA_DEM_mosaic_global_463m>).

**1.3. Crater Counts Statistics**

We performed crater counts statistics at two highland exposures mapped as Early Noachian in the global geologic map of Mars^1^, one located in NW Hellas (Figs. S5) and the other in NW Isidis (Figs. S7). Crater size-frequency distribution plotted in a differential format using the production function of Hartmann & Daubar^2^ and the chronology function of Hartmann^3^ as updated by Michael^4^. For the crater count age determinations in Fig. S5, we counted craters down to ~30 m in diameter. However, we made these age determinations using only craters between ~700 m and ~1 km in diameter. The areas for the counts are ~ 5,000 km^2^. Warner et al.^5^ suggest minimum count areas of ~ 1000 km^2^ for accurate results. For the crater count age determinations in Fig. S7, we counted fresh craters between ~25 and ~300 m over an area of 156 km^2^.

**References cited in this section**

(1) Tanaka, K. L. *et al*. Geologic map of Mars. U.S. Geological Survey Scientific Investigations Map 3292, scale 1:20,000,000,<http://pubs.usgs.gov/sim/3292/> (2014) Date of access: 03/14/2019.

(2) Hartmann, W. K., & Daubar, I. J. Martian cratering 11. Utilizing decameter scale crater populations to study Martian history. *Meteoritics & Planetary Science*, **52**(3), 493-510 (2017).

(3) Hartmann, W. K. Martian cratering 8: Isochron refinement and the chronology of Mars. *Icarus*, **174**(2), 294-320 (2005).

(4) Michael, G. G. Planetary surface dating from crater size-frequency distribution measurements: Multiple resurfacing episodes and differential isochron fitting. *Icarus*, **226**(1), 885-890 (2013).

(5) Warner, N. H., Gupta, S., F., C., Grindrod, P., Boll, N., Goddard, K., Minimum effective area for high-resolution crater counting of Martian terrains. *Icarus*, **245**, 198-240 (2015).

**2. Supplementary Figures**


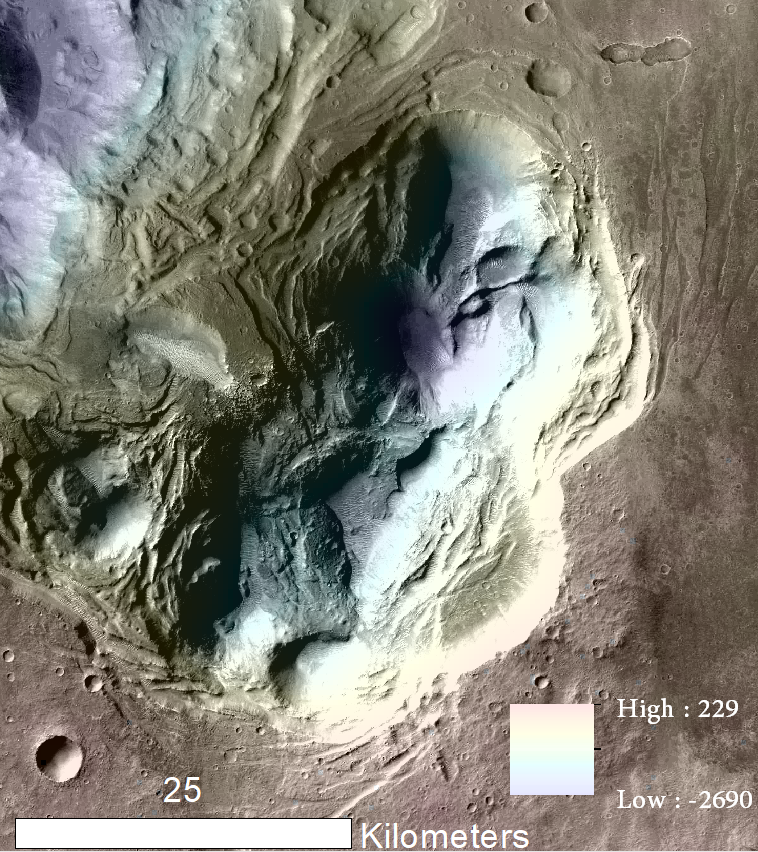
**Fig. S1** View of a potential sinkhole site in a Martian area of widespread chaotic terrain formation (16° 49’24.10 “S, 39° 54’52.66 “W). The sinkhole is strikingly similar to some terrestrial examples in the Dead Sea region (e.g., <https://www.eskp.de/fileadmin/eskp/artikel/naturgefahren/erdfall/Doline_Totes_Meer.jpg> and <https://imaggeo.egu.eu/view/4775/> ). The image is a composite of a color MOLA digital elevation model (460 m/pixel, credit: MOLA Science Team, MSS, JPL, NASA) draped over a CTX mosaic (~6 m/pixel, credit: NASA/JPL. License terms can be found at pds-imaging.jpl.nasa.gov/portal/mro_mission.html).


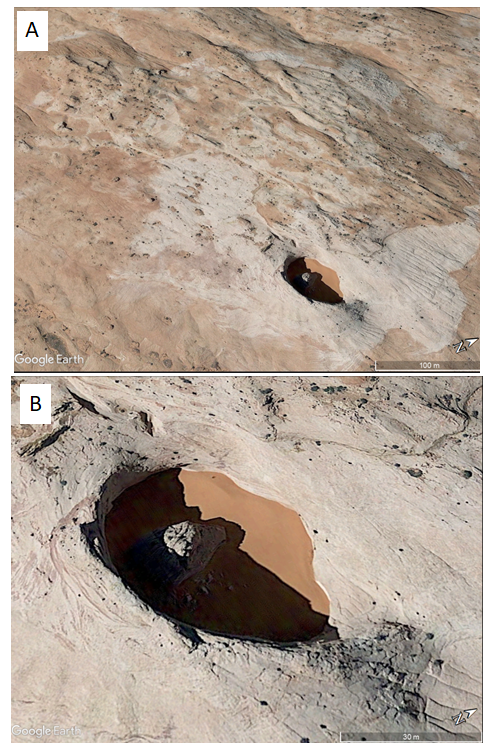


**Fig. S2** View of a wind excavated pit (60 m wide by 20 m deep) with a central mound located at 37° 40’58.60 "N, 111°18'57.11"W. The pit is a case study for the effect of the ability of strong winds and vortices to shape lithiﬁed sedimentary rock over long periods of time, a process that is believed to have formed the moat that surrounds Mount Sharp in Gale Crater [Chan, M. A., & D. I. Netoff. A terrestrial weathering and wind abrasion analog for mound and moat morphology of Gale crater, Mars, *Geophys. Res. Lett*., **44**, 4000–4007, doi:10.1002/ 2017GL072978 (2017)].

**Fig. S3** Views of channels that transition into inverted relief floors in NW and W Hellas. The locations for each panel are: (A) 28°35'17.60"S, 55° 5'46.99"E; (B) 25°43'9.38"S, 51°54'2.18"E; (C) 22°45'27.77"S, 57°56'5.34"E; (D) 34° 7'0.54"S, 35°58'11.55"E; (E) 33°20'23.76"S, 38°12'29.74"E. All panels are parts of a CTX mosaic (6 m/pixel, credit: NASA/JPL. The license terms can be found at pds-imaging.jpl.nasa.gov/portal/mro_mission.html).

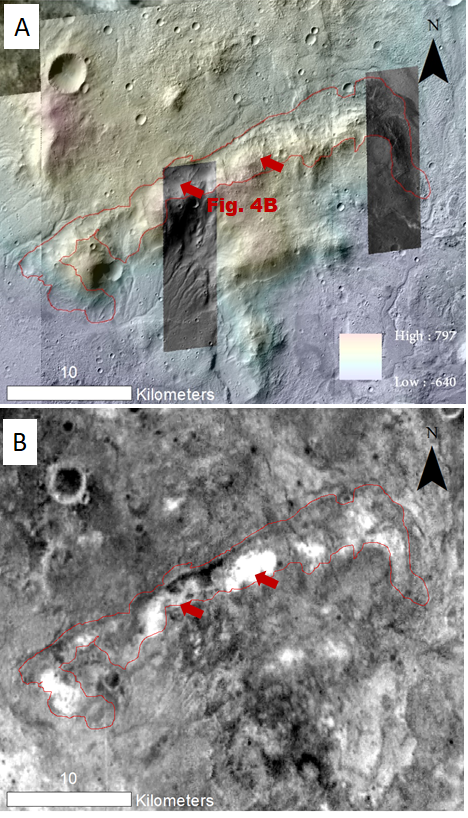


**Fig. S4 (A)** View of Early Noachian promontory shown in Fig. 4 and centered at 23°30’S, 59°37’E. The HiRISE images in the region are superposed, and the location of Fig. 4B is indicated. The base image is a composite of a color MOLA digital elevation model (460 m/pixel, credit: MOLA Science Team, MSS, JPL, NASA) draped over a CTX mosaic (6 m/pixel, credit: NASA/JPL. The license terms can be found at pds-imaging.jpl.nasa.gov/portal/mro_mission.html). **(B)** Red arrows show areas in the promontory affected by relatively deep wind erosion. Note that these areas have bright surfaces in night infrared images, indicating that its surfaces preferentially retained the diurnal heat signature during the night time. This panel is part of a THEMIS nighttime IR (infrared) Global Mosaic (<http://www.mars.asu.edu/data/>, 100 m/pixel, credit: Christensen, P. R. *et al.* THEMIS Public Data Releases, Image Explorer, Planetary Data System node, Arizona State University, <http://themis-data.asu.edu>, (2006) Date of access: 04/28/2019.)


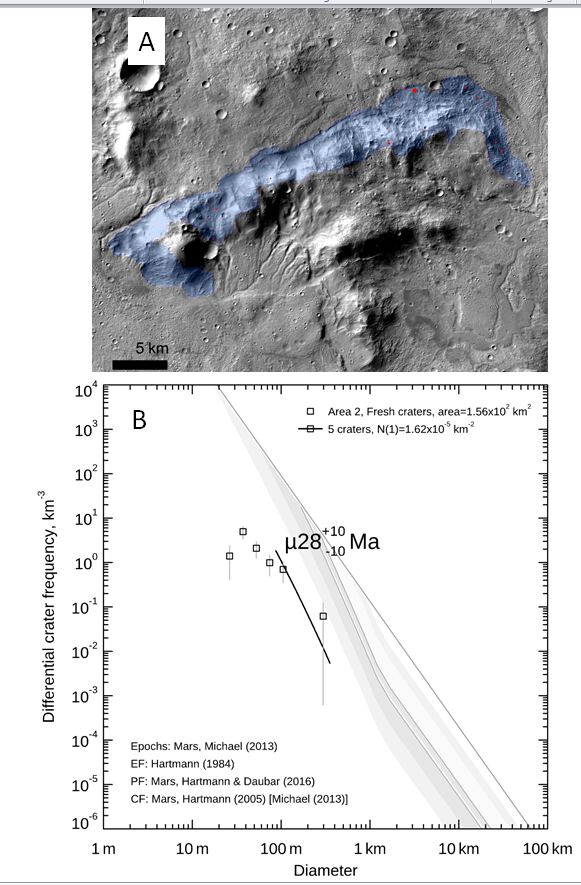


**Fig. S5** Crater counts statistics for **(A)** CTX image mosaic showing the Early Noachian promontory shown in Fig. 4 and centered at 23°30’S, 59°37’E. red marks indicate measurements of crater diameters for fresh, non-disrupted craters within the rim areas (blue), which corresponds to the wind-eroded surfaces on the promontory. **(B)** Plot showing model age of ~28 Ma. The red dots are the counted craters (see. Supplementary Materials on Methods and Datasets). EF stands for Equilibrium Function (the saturation line). PF for production function, CF for chronology function. Gray shaded areas are Epochs as defined by the functions. The mu symbol means that the age is model age.


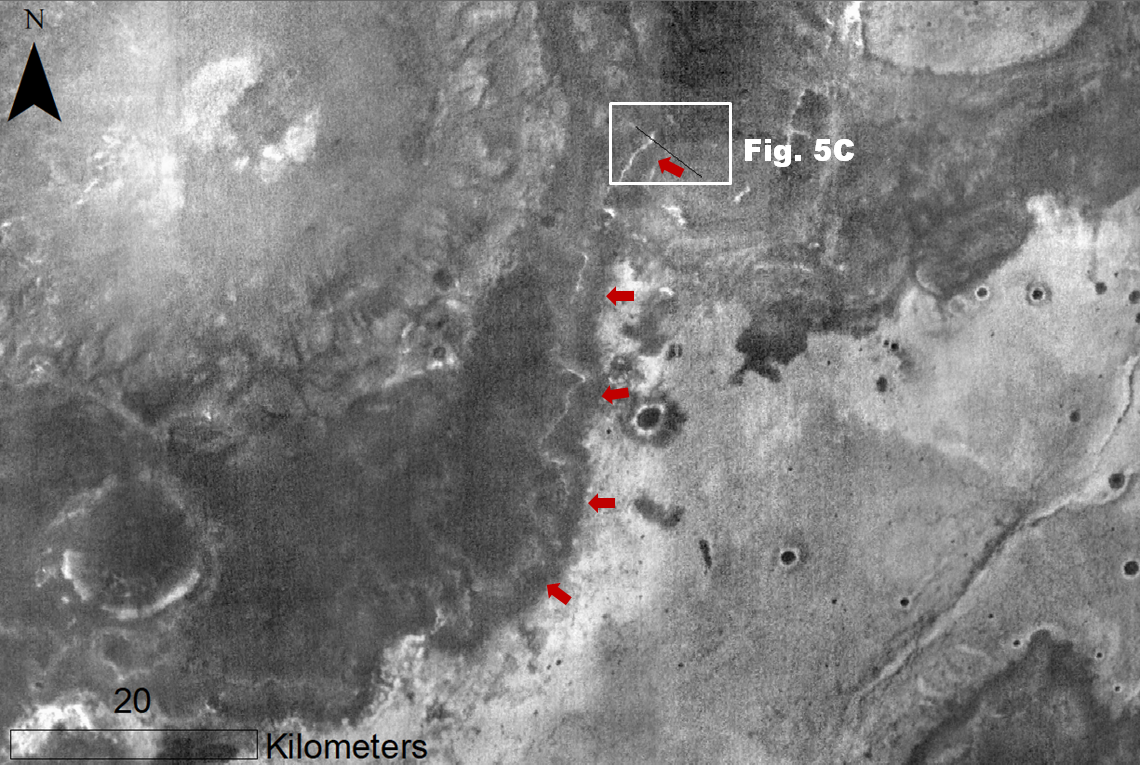


**Fig. S6** Context nighttime infrared view for Fig. 5C. The red arrows indicate the location of the scarp’s talus slopes. Note that they exhibit a dark thermal albedo. This view is part of a THEMIS nighttime IR (infrared) Global Mosaic (<http://www.mars.asu.edu/data/>, 100 m/pixel, credit: Christensen, P. R. *et al.* THEMIS Public Data Releases, Image Explorer, Planetary Data System node, Arizona State University, <http://themis-data.asu.edu>, (2006) Date of access: 04/28/2019.)


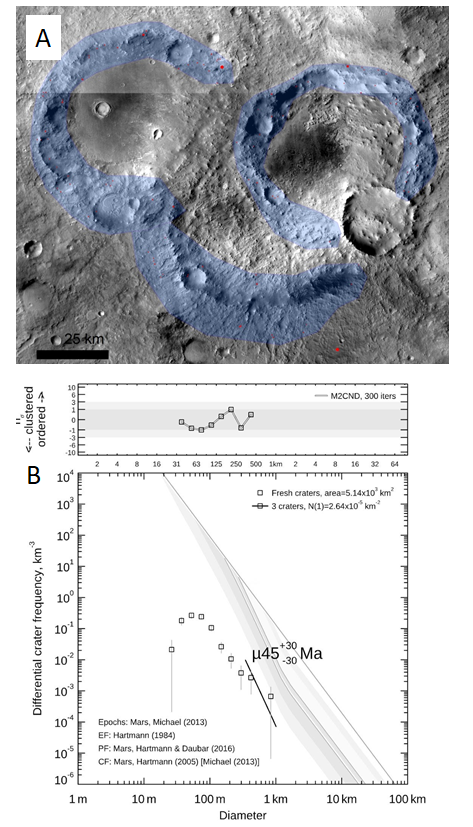


**Fig. S7** (**A)** CTX image mosaic showing three disrupted craters; red marks indicate measurements of crater diameters for fresh, non-disrupted craters within the rim areas (blue). **(B)** Model age shows that disruption ended no more than ~75 Ma in the Late Amazonian. (see. Supplementary Materials on Methods and Datasets). EF stands for Equilibrium Function (the saturation line). PF for production function, CF for chronology function. Gray shaded areas are Epochs as defined by the functions. The mu symbol means that the age is model age.


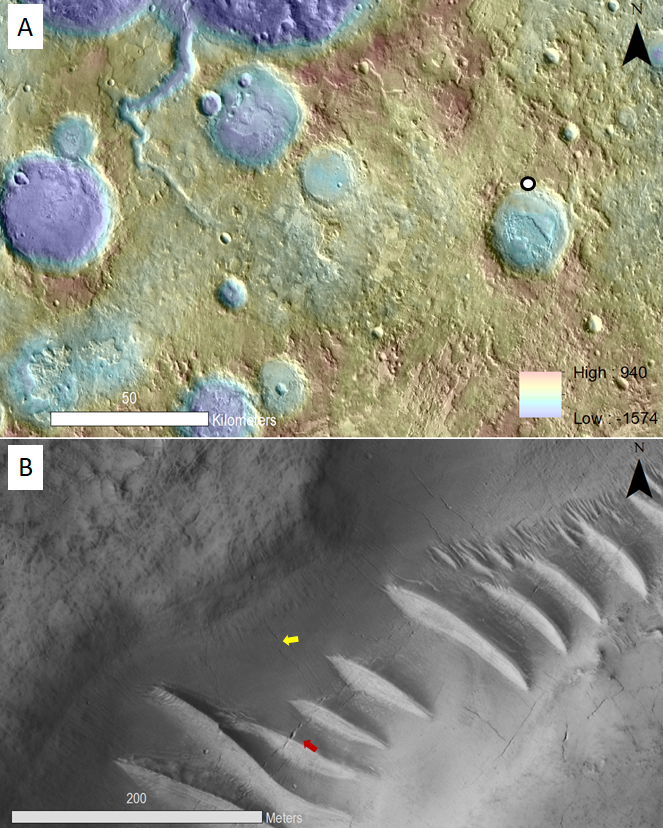


**Fig. S8 (A)** View of an area in NE Arabia Terra (see also Fig 7C) centered at 28°26’N, 63°30’E characterized by significant surface relief losses due to wind erosion. The context for panel B is shown as the white dot. The base image is a composite of a color MOLA digital elevation model (460 m/pixel, credit: MOLA Science Team, MSS, JPL, NASA) draped over a CTX mosaic (6 m/pixel, credit: NASA/JPL. The license terms can be found at pds-imaging.jpl.nasa.gov/portal/mro_mission.html). **(B)** Part of HiRISE image ESP_037159_2090 centered at 29° 5’58’"N, 65° 6’6’"E. The yellow arrow shows talus material, which generally lacks reworking into bedforms. The red arrow shows the location of a fractured dune, indicating that it is a fossil deposit.

**3. Impact Formation of Fines**

Whereas clay-, silt-sized, and sand materials would have been produced and co-deposited in impact basin ejecta blankets, those sedimentary components would have been readily “armored” against aeolian stripping. However, expected explosive jets of gases, solids, and impact melt released from the impact’s transient cavity could have produced the far-field ejecta and disperse material globally for large impact craters and multi-ringed impact basins.

Upon entry back into the atmosphere, coarse jetted material would have fallen at high speeds to the surface, producing a heterogeneous, patchy distal ejecta blanket and secondary craters. Fine materials, however, would have neither ablated, nor impacted rapidly, but would have likely been entrained in the atmosphere and further dispersed. The atmosphere would have winnowed some of the fine materials, such that they settled after the emplacement of the main blocky ejecta blanket.

We note that lunar soil— generally impact-generated — consists dominantly (as assessed by weight fractions) of particle sizes smaller than 1 mm, and much of it is finer than 2 microns^1^. Lorenz^2^ modeled the global dispersal of impact-melted ejecta from a 15-km-size crater on Mars— drawing also from observations of dark impact haloes and parabolas on Venus— and considered this process as likely to have made an observable contribution to Martian soils. Spirit rover observations of soils at Gusev Crater were interpreted as wind-reworked, impact-crushed basaltic rock^3^.

On Mars, fine solid jetted material would have tended to be size-graded (upwards fining) for particles of a size where the airfall descent time through the lower, denser part of the atmosphere (here estimated as the lower one scale height— 11 km) is comparable to or longer than the parabolic transit speed of coarse ejecta in free flight. For example, taking 1 hour as characteristic parabolic transit time for globally distributed ejecta, the particle radius where there is a longer terminal speed settling time through the lower 11 km’ of the atmosphere is around 42 microns radius if the particle density is 3000 kg/m^3^. This calculation ignores some factors, such as the fact that the atmosphere would have been extremely windy in the hour following impact, and atmospheric density and particle settling times may have been much greater. However, it suggests that particles coarser than several tens of microns will tend to co-deposit with ballistic ejecta, and particles a few tens of microns and smaller in radius would have tended to be fining-upwards graded on top of the ballistic ejecta. Due to impact-related atmospheric disturbances, this diameter may have been greater, maybe up to millimeters in radius.

The fine, globally distributed material would be subject to aeolian reworking as dust and sand. If rainfall or snowmelt occurred on this layer due to impact-induced climatic disturbance, the entire airfall unit also would be subject to rapid fluvial reworking.
Not all the jetted material would have been pulverized solids. Some fraction would have been either melted or vaporized depending on impact speeds. Large amounts of dust could have been formed by impact vapor condensation and vapor-driven fragmentation of impact melt. A classic and still partly relevant work was done by Kieffer and Simonds^4^. Their calculations, backed by observations of 32 relatively fresh terrestrial craters, led the authors to a conclusion that impact ejecta dynamics for sedimentary targets are different from impacts into ‘crystalline’ (igneous or low-volatile high-grade metamorphic) rocks. A chief difference is that violent volatile release from sedimentary rocks results in an explosive dispersal of melted rock products, whereas more coherent melt sheets are generated by impacts into crystalline rocks. Kieffer and Simonds^4^ also proposed that flash alteration of silicates to phases, such as clay minerals (phyllosilicates) takes place in superheated ejecta clouds and jets of partly molten and vaporized materials. Thus, the ejecta cloud chemistry could have contributed to the accumulation of soft mineral types prone to subsequent aeolian erosion.

A model of the Meteor Crater-forming bolide impact of Canyon Diablo in Arizona, USA, led to similar conclusions to those presented in Kieffer and Simonds, but in more detail^5^. Their observations and model found that over 80% of the ‘iron’ bolide melted and explosively dispersed by CO_2_ released from the impacted Kaibab formation (limestone). Much of these materials spread far around the crater as sand-size and finer sediments.

Most likely, impact-melt dust was spread through the atmosphere even more distantly and diffusely, maybe a regional equivalent of the global K/T boundary Chicxulub ejecta. In the latter example, a layer of iridium-rich clays and local 6-cm-thick impact melt spherule bed, totaling an average of 35 cm thick, was found 2500 km from the crater^6^. If we consider the transient crater diameters and depths of the Chicxulub versus Hellas impact, the 2500 km distant ejecta for Hellas might be 100 m thick, including 18 m of impact melt spherules— all other things equal.

**References cited in this section**

(1) McKay, D.S. *et al.* The Lunar Regolith, Chapter 7 in Heiken G. H, Vaniman D. T. & French B. M., eds. *The Lunar Sourcebook*, Cambridge University Press, New York NY, pp. 285-356 (1991).

(2) Lorenz, R. Microtektites on Mars: Volume and Texture of Distal Impact Ejecta Deposits. *Icarus*, **144**, 353–366, doi:10.1006/icar.1999.6303 (2000)

(3) McGlynn, I.O., Fedo, C.M. & H.Y. McSween Jr. Origin of basaltic soils at Gusev crater, Mars, by aeolian modification of impact‐generated sediment. *Jour. Geophys. Res.* **116**, E00F22, doi:10.1029/2010JE003712 (2011).

(4) Kieffer, S.W. & C.H. Simonds, *Rev. Geophys. Space Phys.* **18**, 143-181 (1980).

(5) Schnabel, C. E. *et al.* Shock Melting of the Canyon Diablo Impactor: Constraints from Nickel-59 Contents and Numerical Modeling. *Science* **285** (5424), 85-88. DOI: 10.1126/science.285.5424.85 (1999).

(6) Esmeray-Senlet, S., K.G. Miller, R.M. Sherrell, T. Senlet, J. Vellekoop & H. Brinkhuis. Iridium profiles and delivery across the Cretaceous/Paleogene boundary. *Earth Planet. Sci. Lett.* **457**, 10.1016/j.epsl.2016.10.010 (2016).

**4. Glacial Formation of Silt**

On Earth, glaciers are probably the largest source of aeolian silt^1^ and enormous volumes of sand. For example, in China, the silt and clay of the Loess Plateau (area 440,000 km^2^, volume ~50,000 km^3^ (2,3)) and the related Taklamakan Desert sand sea (330,000 km^2^, roughly 300,000 km^3^ (4)) were derived by fluvial and aeolian winnowing mainly from glacio-alluvial and glacio-lacustrine sediments^2^.

Glacigenic silt with minor components of clay and fine sand represent an important compositional makeup of central China’s Loess Plateau^5-13^. Aeolian winnowing of dunes and interdune playas in the Taklamakan Desert^9^ and Tibetan glacier-fed evaporative lakes^10, 11^ are thought to have constituted a major sedimentary supply to the Loess Plateau. The formation of the loess deposit has been gradual and linked to paleoclimatically controlled fluxes in wet-based temperate and polythermal glaciers in the Kunlun Shan, Pamirs, and Karakoram mountains since the Late Miocene^12^. During the current interglacial, another major source of dust in the Loess Plateau is the influx of non-glacial Gobi Desert dust^13^. On Mars, glacial climatic epochs may have similarly generated large amounts of silt and fine sand, which then may have been reworked during periods of impact-induced climatic instability.

**References cited in this section**

(1) Derbyshire, E. & L.A. Owen. Glacioaeolian Processes, Sediments, and Landforms. Chapter 8 in: *Past Glacial Environments*. DOI: <http://dx.doi.org/10.1016/B978-0-08-100524-8.00008-7> (2018).

(2) Rittner, M., P. *et al*. The provenance of Taklamakan desert sand. *Earth and Planet. Sci. Lett.* 437, 127-137 (2106).

(3) Xiao, G. *et al*. Spatial and glacial-interglacial variations in provenance of the Chinese Loess Plateau. *Geophys. Res. Lett.* 39, L20715, doi:10.1029/2012GL053304 (2012).

(4) Zhu, Y., X. Jia, & M. Shao. Loess Thickness Variations Across the Loess Plateau of China. *Survey in Geophysics* 39, 715-727 (2018).

(5) Chang, Q., Mishima, T., Yabuki, S., Takahashi, Y., & H. Shimuzu. Sr and Nd isotope ratios and REE abundances of moraines in the mountain areas surrounding the Taklimakan Desert, NW China. *Geochemical Journal*, **34**, 407-427 (2000).

(6) Pullen, A. *et al*. The Qaidam Basin and northern Tibetan Plateau as dust sources for the Quaternary Chinese Loess Plateau and paleoclimatic implications. *Geology*,. **39**, 1031-1034. 10.1130/G32296.1 (2011).

(7) He, T., Liu, L., Chen, Y., Sheng, X. & J. Ji. A seven-million-year hornblende mineral record from the central Chinese Loess Plateau, *Scientific Reports*, **7**, Article Number 2382 (2017).

(8) Clift, P.D. *et al*. Controls on erosion in the western Tarim Basin: Implications for the uplift of northwest Tibet and the Pamir: *Geosphere*, **13** (5), 1747–1765, doi:10.1130/GES01378.1 (2017).

(9) Wang, X., Dong, Z., Zhang, J. & G. Chen. Geomorphology of sand dunes in the Northeast Taklimakan Desert. *Geomorphology*, **42**, 183– 195 (2002).

(10) Chen, F., Qiang, M., Zhou, A., Xiao, S., Chen, J. & D. Sun. A 2000-year dust storm record from LakeSugan in the dust source area of arid China. *J. Geophys. Res. Atmos.*, **118**, 2149–2160, doi:10.1002/jgrd.50140 (2013).

(11) Dietze, E. *et al*. Sediment transport processes across the Tibetan Plateau inferred from robust grain-size end members in lake sediments, *Clim. Past*, **10**, 91–106, [www.clim-past.net/10/91/2014/](http://www.clim-past.net/10/91/2014/) doi:10.5194/cp-10-91-2014 (2014).

(12) Sun, Y. *et al.* Astronomical and glacial forcing of East Asian summer monsoon variability.*Quaternary Science Reviews*, **115**, 132e142 (2015).

(13) Wei, T., Dong, Z., Kang, S. & S. Ulbrich. Tracing the Provenance of Long-Range Transported Dust Deposition in Cryospheric Basins of the Northeast Tibetan Plateau: REEs and Trace Element Evidences. *Atmosphere*, **9**, 461; doi:10.3390/atmos9120461 (2018).

**5. The Production of Impact Melt Associated with the Formation of Hellas**

In the case of the Hellas impact, we further examine the amount of impact melt likely generated by the event. Key factors controlling the amount of melt (and vapor) produced by this means are the impactor’s size, estimated impact speed, and a relationship between melt and vapor production with impact speed. The Hellas impactor is variously estimated to have had a radius between 75 km and 300 km^1, 2^. The range of possible sizes is estimated as a function of both impactor speed and composition, Hellas basin’s transient cavity size, and the geometric properties and rock structure of the model target. Impact speed could have ranged from Mars escape speed of 5 km/s up to ~25 km/s, but most asteroid impact speeds at Mars are in the range of 10-14 km/s (3). Within the narrower more-probable range, the mass of impact melted and vaporized material ranges from about 0.1 to 3 times the projectile mass for stony (basalt or dunite) projectiles and targets^2^ — the lower yield being for 10 km/s, and the higher one for 14 km/s impact speeds. We consider two cases: an impactor of radius 125 km and impact speed 10 km/s, or 100 km and 14 km/s. In the first case, with the vapor and melt yield given by Quintana et al.^4^, we estimate a total volume of impact melt plus vapor (condensed nonporous volume equivalent) of 818,000 km^3^; and 12,560,000 km^3^ for the larger yield. Based on Zhu et al.^5^, we estimate the volume of ejecta produced by Hellas to be 13,225,000 km^3^ [(10 km * 1,150 km (Rb, basin radius) *1,150 km)]. By comparison, the Deccan Traps, Earth's largest accumulation of flood basalts, have an approximate volume of 1,000,000 km^3^ (6). Approximately 30-50% of the Hellas ejecta would have been distributed beyond 2 Rb (i.e., beyond continuous ejecta blanket). Based on their approach, 30% of the ejecta (3,967,500 km^3^) could have been melt. By comparison, the Mediterranean Sea has a volume of 3,750,000 km^3^.

**References cited in this section**

(1) Bierhaus, M., K. Wünnemann, Elbeshausen, D. & G.S. Collins, Numerical modeling of basin-forming impacts on Mars: Implications for the heat budget of planetary interior, 42nd Lunar and Planetary Science Conference 2011, abstract 2128 (2011).

(2) Louzada, K.L. & S. Stewart. Effects of planet curvature and crust on the shock pressure field around impact basins. *Geophys. Res. Lett.* 36, L15203 (2009).

(3) QinNesvorny, D., F. Roig, & W.F. Bottke. Modeling the historical flux of planetary impactors, The Astronomical Journal 153:103. <https://doi.org/10.3847/1538-3881/153/3/103> (2017).

(4) Quintana, S.N., Crawford, D.A. & P.H. Schultz. Analysis of impact melt and vapor production in CTH for planetary applications, The 13th Hypervelocity Impact Symposium, Procedia Engineering **103**, 499-506 (2015).

(5) Zhu, M. H., Wünnemann, K., & Artemieva, N. Effects of Moon’s thermal state on the impact basin ejecta distribution. *Geophysical Research Letters*, **44**, 11,292–11,300. <https://doi.org/10.1002/2017GL075405> (2017).

(6) Dessert, C. et al. Erosion of Deccan Traps determined by river geochemistry: impact on the global climate and the 87Sr/86Sr ratio of seawater. Earth and Planetary Science Letters. **188** (3–4): 459–474. [Bibcode](https://en.wikipedia.org/wiki/Bibcode_(identifier)):[2001E&PSL.188..459D](https://ui.adsabs.harvard.edu/abs/2001E&PSL.188..459D). [doi](https://en.wikipedia.org/wiki/Doi_(identifier)):[10.1016/S0012-821X(01)00317-X](https://doi.org/10.1016%2FS0012-821X%2801%2900317-X). (2001).

**6. A Case for the Future in-situ Astrobiological Exploration: Was the Early Noachian Fine-grained Crust an Important Source of Mud to the Northern plains Mud Volcanoes?**

The northern plains of Mars contain tens of thousands of potential mud volcanoes^1-5^. A proposed possible origin is through rapid emplacement and compaction of vast volumes of fine-grained sediments that were discharged into the northern plains by catastrophic floods^4^. This hypothesis is particularly relevant to mud volcanoes located within the sedimentary units covering the depocenters of the northern plains interior basins^4^. However, since many mud volcanoes occur along the margins of these basins (i.e., in areas where the northern plains materials form relatively thin deposits)^5^, the location of the source mud-rich stratigraphy remains uncertain.

The identification of numerous quasi-circular depressions throughout the northern plains indicates the presence of a densely cratered buried surface^6,7^. Crater counts statistics indicate that this surface is of Early Noachian age^7^. Spectral analyses of some of its outcropping exposures reveal that these materials include phyllosilicates that are similar to those exposed at widespread highland locations^8^. Hence, it is conceivable that the cratered lowlands and highlands shared similar geologic histories during the Early Noachian.

However, a major difference in the geologic evolution of these terrains is that the burial of the lowland cratered surfaces beneath the northern plains would have effectively increased the thickness of the cryosphere throughout the northern plains. The thickening of the cryosphere, in turn, would have raised the boundary between the hydrosphere and the cryosphere. These subsurface conditions probably permitted the presence of liquid water within cratered lowland substrates, even after the planet turned cryogenic at the boundary between the Late Noachian and Early Hesperian.

Here, we hypothesize that the fine-grained upper crustal highland deposits documented in this article might have extended into the lowlands. Zones within these materials, which existed at sufficient depths to have contained liquid water, could have evolved to become geologic source regions of some of the northern plains mud volcanoes. These “wet” deposits, if present within the cratered lowland stratigraphy, would be ideal mud volcano sources because they would have been comprised of thick, massive, sequences of highly sorted dust-sized particles.

The figure below is part of a CTX mosaic that shows a cluster of mud volcanoes (15°11'16.32"N, 85°11'15.28"E) occurring within a few hundred kilometers from the NE Arabia Terra region that we document in this article.


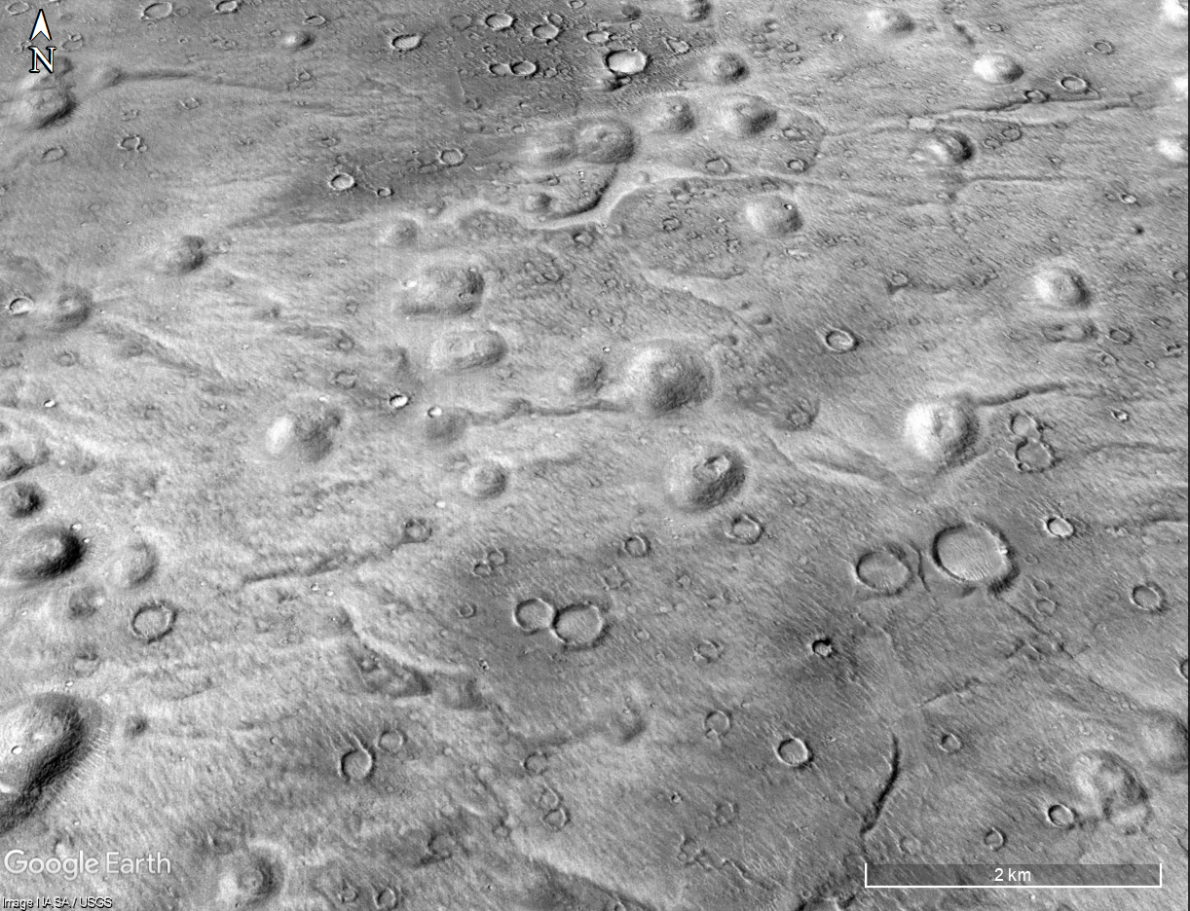


Within the above hypothetical context, northern plains mud volcanism would have extruded significant volumes of some of the oldest sediments of Mars from potentially habitable environments. However, because these materials could be ~4 Ga or older, it would be essential to develop an approach for detecting the biosignatures with the greatest longevity through geologic time, such as lipids^9, 10^. Wilhelm et al.^11^ have proposed a life-detection mission concept to search for these robust molecular biosignatures in northern plains mud volcanoes where optimal organic accumulation and preservation has potentially occurred.

**References cited in this section**

(1) Tanaka, J. Sedimentary history and mass flow structures of Chryse and Acidalia Planitiae, Mars. *Geophys. Res.*102, 4131–4150 (1997).

(2) Farrand, W. H., L. R. Gaddis, and L. Keszthelyi , Pitted cones and domes on Mars: Observations in Acidalia Planitia and Cydonia Mensae using MOC, THEMIS, and TES data, *J. Geophys. Res.*,110, E05005 doi:10.1029/2004JE002297 (2005).

(3) Skinner, J. A., and K. L. Tanaka. Evidence for and implications of sedimentary diapirism and mud volcanism in the southern Utopia highland-lowland boundary plain, Mars, Icarus, 186, 41–59 doi:10.1016/j.icarus.2006.08.013 (2007).

(4) Oehler and Allen. Evidence for pervasive mud volcanism in Acidalia Planitia, Mars,  Icarus 208, 636-657 (2010).

(5) Rodriguez, J. A. P., *et al*. Infiltration of Martian outflow channel floodwaters into lowland cavernous systems. *Geophys. Res. Lett.*, 39, L22 201 doi:10.1029/2012GL053225 (2012).

(6) Buczkowski, D. L., Frey, H. V., Roark, J. H. & McGill, G. E. Buried impact craters: A topographic analysis of quasi-circular depressions, Utopia Basin, Mars. *J. Geophys. Res.* **110** (2005).

(7) Frey, H. V. Impact constraints on, and a chronology for, major events in early Mars history. *J. Geophys. Res.* **111**(E8)<https://doi.org/10.1029/2005je002449> (2006).

(8) Carter, J., Poulet, F., Bibring, J. P. & S. Murchie. Detection of Hydrated Silicates in Crustal Outcrops in the Northern Plains of Mars. *Science*, **25**, 328, 1682-1686 DOI: 10.1126/science.1189013 (2010).

(9) Eigenbrode, J. L. Fossil lipids for life-detection: a case study from the early Earth record. In Strategies of Life Detection (pp. 161-185) Springer, Boston, MA. (2008).

(10) Wilhelm, M. B., *et al.* Xeropreservation of functionalized lipid biomarkers in hyperarid soils in the Atacama Desert. Organic geochemistry, 103, 97-104. (2017).

(11) Wilhelm, M. B. *et al.* Abzu: A Mission to Uncover the Origin of Organic Material on Mars. In AGU Fall Meeting 2019 (2019).
